# Hyperexpansion of genetic diversity and metabolic capacity of extremophilic bacteria and archaea in ancient Andean lake sediments

**DOI:** 10.1186/s40168-024-01878-x

**Published:** 2024-09-17

**Authors:** María Ángeles Lezcano, Till L. V. Bornemann, Laura Sánchez-García, Daniel Carrizo, Panagiotis S. Adam, Sarah P. Esser, Nathalie A. Cabrol, Alexander J. Probst, Víctor Parro

**Affiliations:** 1 Centro de Astrobiología (CAB), CSIC-INTA, 28850 Torrejón de Ardoz, Madrid, Spain; 2 IMDEA Water Institute, Avenida Punto Com 2, 28805 Alcalá de Henares, Madrid, Spain; 3 Environmental Metagenomics, Research Center One Health Ruhr of the University Alliance Ruhr, Faculty of Chemistry, University of Duisburg-Essen, Essen, Germany; 4 Centre of Water and Environmental Research (ZWU), University of Duisburg-Essen, Essen, Germany; 5 Institute of General Microbiology, Kiel University, Kiel, Germany; 6 SETI Institute, 339 Bernardo Avenue, Suite 200, Mountain View, CA 94043 USA

**Keywords:** Microbial metabolism, Microbial dark matter, Genome-resolved metagenomics, Lipid biomarkers, Ancient sediments, Andean Altiplano, Mars

## Abstract

**Background:**

The Andean Altiplano hosts a repertoire of high-altitude lakes with harsh conditions for life. These lakes are undergoing a process of desiccation caused by the current climate, leaving terraces exposed to extreme atmospheric conditions and serving as analogs to Martian paleolake basins. Microbiomes in Altiplano lake terraces have been poorly studied, enclosing uncultured lineages and a great opportunity to understand environmental adaptation and the limits of life on Earth. Here we examine the microbial diversity and function in ancient sediments (10.3–11 kyr BP (before present)) from a terrace profile of Laguna Lejía, a sulfur- and metal/metalloid-rich saline lake in the Chilean Altiplano. We also evaluate the physical and chemical changes of the lake over time by studying the mineralogy and geochemistry of the terrace profile.

**Results:**

The mineralogy and geochemistry of the terrace profile revealed large water level fluctuations in the lake, scarcity of organic carbon, and high concentration of SO_4_^2-^-S, Na, Cl and Mg. Lipid biomarker analysis indicated the presence of aquatic/terrestrial plant remnants preserved in the ancient sediments, and genome-resolved metagenomics unveiled a diverse prokaryotic community with still active microorganisms based on in silico growth predictions. We reconstructed 591 bacterial and archaeal metagenome-assembled genomes (MAGs), of which 98.8% belonged to previously unreported species. The most abundant and widespread metabolisms among MAGs were the reduction and oxidation of S, N, As, and halogenated compounds, as well as aerobic CO oxidation, possibly as a key metabolic trait in the organic carbon-depleted sediments. The broad redox and CO_2_ fixation pathways among phylogenetically distant bacteria and archaea extended the knowledge of metabolic capacities to previously unknown taxa. For instance, we identified genomic potential for dissimilatory sulfate reduction in Bacteroidota and α- and γ-Proteobacteria, predicted an enzyme for ammonia oxidation in a novel Actinobacteriota, and predicted enzymes of the Calvin–Benson–Bassham cycle in Planctomycetota, Gemmatimonadota, and Nanoarchaeota.

**Conclusions:**

The high number of novel bacterial and archaeal MAGs in the Laguna Lejía indicates the wide prokaryotic diversity discovered. In addition, the detection of genes in unexpected taxonomic groups has significant implications for the expansion of microorganisms involved in the biogeochemical cycles of carbon, nitrogen, and sulfur.

Video Abstract

**Supplementary Information:**

The online version contains supplementary material available at 10.1186/s40168-024-01878-x.

## Background

Culture-independent tools such as metagenomics have made it possible to decipher the identity and potential metabolisms of a large fraction of the Earth’s microbiome that remains uncultivable, commonly referred to as microbial dark matter [1]. Microbial dark matter is estimated to cover over 99% of microorganisms worldwide [2], and extreme environments for life presumably harbor a large proportion [3]. Microbial communities in extreme environments can be adapted to harsh environmental conditions, in most cases combining low or high temperature [4–6], dryness and/or high UV radiation [6, 7], acidic or alkaline water [8–10], hypersalinity [11, 12], and/or high metal content [8, 9]. Unraveling the uncultured microbial fraction in extreme habitats may open opportunities for biotechnological applications [13] and may expand the theory on the limits of life on Earth and beyond.

High-altitude lakes in the arid and volcanic region of the Altiplano (Chile, Perú, Bolivia, and Argentina) are unique extreme aquatic environments with a poorly explored microbial diversity. A representative case is the Laguna Lejía, a saline, endorheic, groundwater-fed lake located at 4325 m.a.s.l. in the Chilean Altiplano [14]. Laguna Lejía has undergone rapid desiccation caused by current low precipitation (<200 mm year^-1^) and high evaporation rates (1500 mm year^-1^) [15]. As a consequence, the lake water surface has shrunk from ~10 to ~2 km^2^, leaving an extensive sediment outcrop (or terrace) indicating a water level 25 times higher in the late Pleistocene/early Holocene (~10.8–9.2 kyr BP) than today, and strong water level fluctuations over thousands of years [16–18]. In addition, water and sediment chemistry of Laguna Lejía has been influenced by the deposition of pyroclastic material from periodic eruptions of the Lascar volcano, located 5 km to the north [19]. As a result of its hydrologic and climatic history and volcanic influence, Laguna Lejía can be considered as a compelling terrestrial analog of Martian paleolakes (e.g*.*, Jezero crater) [14], which have been suggested as potentially habitable before they underwent rapid evaporation due to a climate change ~3 Ga ago [20].

The fluctuations in the water level and chemistry of Laguna Lejía over millennia may have influenced the microbial genetic and metabolic diversity. The microbial communities inhabiting the water have been exposed to increasing salinity, while those thriving in the sediments in contact with atmospheric conditions have been also exposed to desiccation, strong daily thermal oscillations, and high UV radiation [14]. Previous studies on microbiomes from Laguna Lejía water and sediments are scarce and so far revealed Bacteroidetes (here Bacteroidota), Proteobacteria, and Firmicutes as the most dominant phyla, as well as a variable proportion (6–8%) of unidentified taxa [21, 22]. Recent efforts have been made to shed light on the proportion of unknown microorganisms by bacterial isolation and proteomic characterization [23]. However, it is barely known the taxonomy of these microorganisms below the phylum level and the metabolic capacity they have in this unique environment.

Among Laguna Lejía habitats, the ancient sediments of the terrace might have recorded the most relevant microbial community changes over thousands of years, as it has been at the forefront of the lake water level fluctuations. Microbial communities can adjust quickly to environmental disturbances by shifting their composition and relative abundance toward those populations with functional traits fitting to the new environment [24]. Alternatively, microorganisms can evolve (originating genomic changes) acquiring new functional traits that allow adaptation [25], especially over long timescales [26]. The microbiome and geochemistry of the ancient sediments of the Laguna Lejía terrace have not been investigated, yet they constitute an important legacy of the past ecological conditions of the lake.

In this work, we tested five hypotheses in the Laguna Lejía terrace: (i) the fluctuations and disconnection of the water table provided new ecological niche opportunities for microbial communities, resulting in a different microbiome from that of the wet sediments; (ii) the prokaryotic community structure along the vertical profile of the terrace differs between sediment layers, each corresponding to a specific period of time; (iii) despite the age of the sediments, the terrace currently contains metabolically active extremophilic microorganisms; (iv) the microorganisms on the terrace possess metabolic traits adapted to the extreme geochemistry of the lake; and (v) the terrace harbor novel microorganisms, as it is a poorly explored extreme environment in a remote location. To test these hypotheses, we investigated the biogeochemistry of the Laguna Lejía terrace using a multidisciplinary approach that combines shotgun metagenomics, lipid biomarkers, geochemistry, and mineralogy. To address these questions, we (a) characterized the mineralogical and geochemical (ions, stable isotopes, and lipid biomarkers) environment of the Laguna Lejía terrace, as well as dated the sediments along its vertical profile, (b) explored the composition, relative abundance, and metabolic potential of the bacterial and archaeal communities along the sediment profile using genome-resolved metagenomics, and (c) estimated in silico genome replication indices. The results contributed to unravel the extent of microbial diversity in the Laguna Lejía and expand metabolic potential to unexpected microbial taxa. In addition, the results contributed to understand and forecast the fate of microbial communities in inland aquatic systems impacted by climate change (e.g., global warming), as well as the impact of a rapid climate change on hypothetical microbial life in Martian paleolakes.

## Methods

### Sampling

**Fig. 1:**
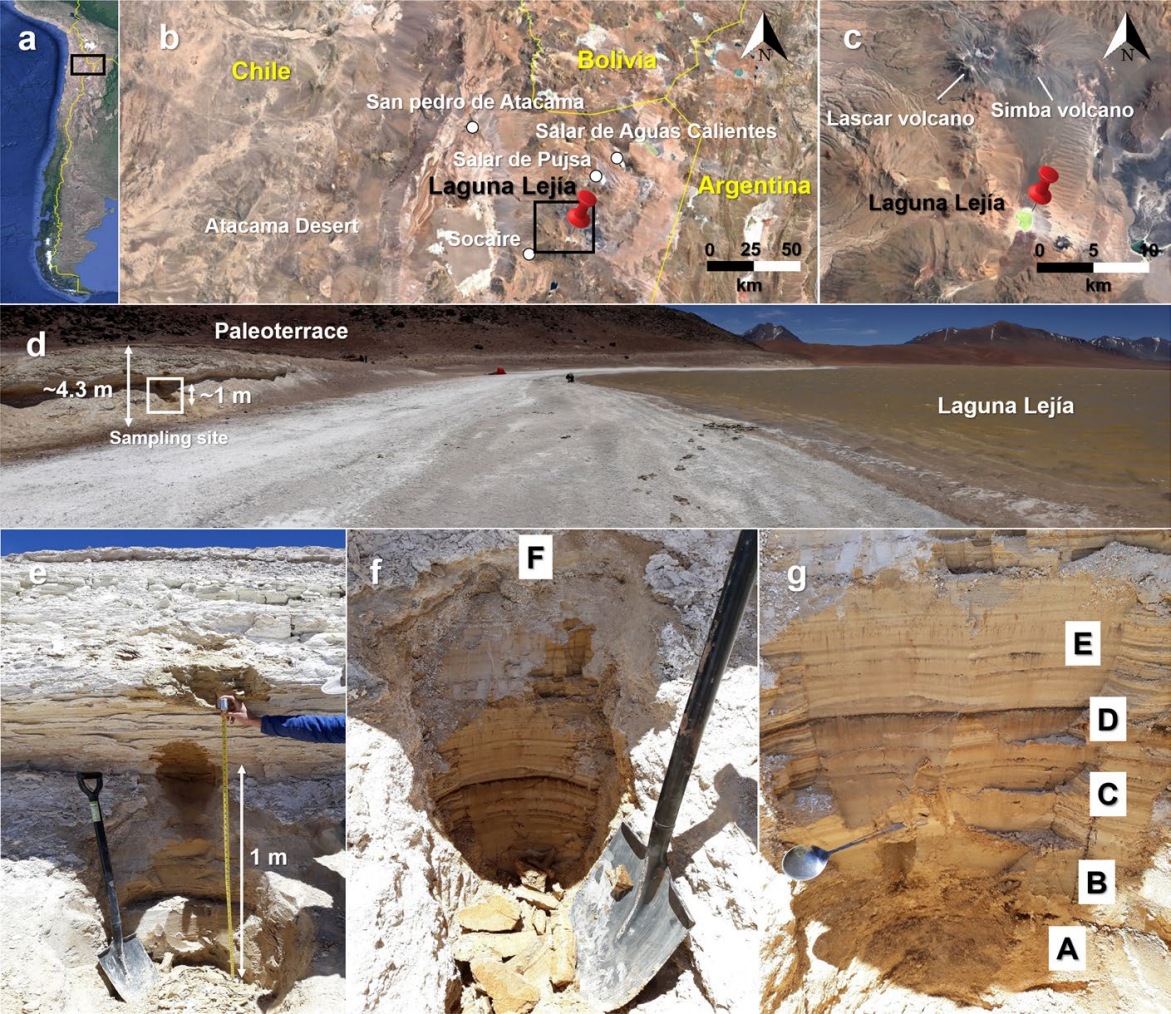
Study site and sampling area located in the Altiplano–Puna Plateau in the Central Andes. **a** and **b** show a sequential zoom of the location of Laguna Lejía (red pin) in northern Chile, where the Altiplano–Puna Volcanic Complex (APVC) is located. **c** shows the endorheic basin of Laguna Lejía surrounded by the active volcanoes Lascar and Simba. **d** shows the panoramic view of Laguna Lejía showing the terraces and the dry shore due to water retreat over the years. The white box indicates the location of the sampled sediment profile, located the first meter above the base of the terrace. **e** shows the ~1-m high vertical profile sampled. **f** and **g** zoom in the sampled profile and show the six sediment layers collected from the bottom (A) to top (F) based on their different color and texture. Starting from the first meter of the terrace, samples were collected from 1 to 1.07 m (A), 1.08 to 1.09 m (B), 1.10 to 1.35 m (C), 1.36 to 1.38 m (D), 1.39 to 1.70 m (E) and 1.71 to 1.95 m (F) high. Pictures **a**, **b**, and **c** are from Google Earth.

Laguna Lejía in the Chilean Andes (4325 m.a.s.l.; 23° 30’ S, 67° 42’ W) (Fig. 1a–c) was sampled during a field campaign in November 2018. Sediment samples were collected from a 1-m high stratified sediments belonging to a 4-m high lake terrace located 10 m away from the current lake shoreline (Fig. 1d). The 1-m sampled section started at 1-m high from the base of the outcrop (Fig. 1d and e) and covers two of the lithologic phases described in previous works in the lake terrace [16, 18]. The lower half of the profile consisted of alternating beds of bentonite, diatomite, and sand (unit II), and the upper half consisted of sand, carbonates, diatomite, and a prominent clastic layer with gravel (unit III) [16, 18].

Before sampling, we removed the exposed face of the outcrop sediments (ca. 10 cm thick) with an ethanol-cleaned shovel to collect samples only from the interior and make sure to avoid the most recent external material. Six horizontal sediment layers were sampled at different depths according to different colors and textures using a clean spoon wiped with ethanol between samples (Fig. 1f and g). Samples were named as A (bottom; 0–7 cm), B (7–9 cm), C (9–35 cm), D (35–38 cm), E (38–70 cm), and F (top; 70–95 cm), and stored in 100-mL polyethylene bottles at 4°C until arrival to the laboratory (~5 days). Then, a fraction of ~100 g of each sample was stored at −80°C and lyophilized for downstream analysis. The remainder of the samples were stored at −20°C.

### Radiocarbon dating

A total of 20 mg of fresh (wet weight) sample A (bottom; 0–7 cm), C (middle depth; 9–35 cm), and F (top; 70–95 cm) were used for radiocarbon analysis on a ^14^C-Accelerator Mass Spectrometry (AMS) dating (Beta Analytic, Inc., Miami, FL, USA). A pre-treatment consisting of acid washes with HCl was applied to decarbonate the samples. Afterwards, ^14^C results were obtained from the total organic content of the sediments. The analysis was conducted using the BetaCal 4.2 program and the high probability density range method [27], along with the IntCal20 database [28].

### Geochemical analysis

In the six samples (A–F), soluble anions, specifically nitrate (NO_3_^-^), nitrite (NO_2_^-^), phosphate (PO_4_^3-^), sulfate (SO_4_^2-^), chloride (Cl^-^), fluoride (F^-^), and bromide (Br^-^), were determined in triplicates by ion chromatography (IC). One gram of dry sediment was suspended in 10 mL of IC-grade water, vortexed for 2 min, and incubated with agitation overnight. Samples were filtered through 0.22-µm PTFE filters (Sartorius, Göttingen, Germany) to remove mineral particles and 5 mL were used for analysis in a Metrohm 861 Advanced Compact Ion Chromatographer (Metrohm AG, Herisau, Switzerland) using two serial dilutions (1:1 and 1:10). Chromatographic separation of ions was performed in a Metrosep A supp 7–250 column (Metrohm AG) with a mobile phase that consisted of 3.6 mM sodium carbonate with a flow rate of 0.7 mL·min^-1^. Anion quantification was performed with a 6-point calibration curve from each commercial anion standard (Sigma-Aldrich, St. Louis, MO). The concentration of elements (e.g., Nitrogen) was calculated as the sum of the elemental concentration of the different anion species (e.g*.*, NO_2_^-^ and NO_3_^-^). Since nitrite (NO_2_^-^) was below quantification limit (<1.6 ppb), the elemental concentration of nitrogen was calculated considering nitrate alone (i.e., NO_3_^-^-N).

Elemental analysis of the cationic soluble fraction was determined in triplicates with the remaining 5 mL of the previous filtered solution of the sediment using inductively coupled plasma mass spectrometry (ICP-MS). A semiquantitative analysis was performed using a PerkinElmer NexION 2000 ICP-MS instrument (PerkinElmer Inc.) with 47 elements used as external standards (Table S1) following the Method 3052 from the Environmental Protection Agency [29].

pH was measured in triplicates in a suspension of substrate:IC-grade water (1:2.5) with a Eutech pH700 Meter (Thermo Fisher Scientific, Waltham, MA, USA).

### Mineralogical analysis

Mineralogical characterization of the six powdered samples was performed with X-ray diffraction (XRD). XRD profiles were obtained with a Bruker D8 Eco Advance diffractometer (Bruker AXS GmbH, Germany) using Cu Kα radiation (*λ* = 1.5406 Å) and a Lynxeye XE-T linear detector. Samples were scanned from 5° to 60° (2Ɵ) with a step size of 0.05° and a step duration of 1 s. The X-ray generator was operated at 40 kV and 25 mA. Phase identification was achieved by comparing the measured diffraction patterns (diffractograms) with those of the PDF-2 Database using the DIFFRAC.EVA software (Bruker AXS).

### Bulk geochemistry analysis

The six lyophilized samples were homogenized using a mortar and pestle. Stable isotope composition of organic carbon (δ^13^C) and total nitrogen (δ^15^N) was measured by isotope-ratio mass spectrometry (IRMS) using a MAT 253 (Thermo Fisher Scientific, Waltham, MA, USA), following analytical methods by the U.S. Geological Survey [30], as described elsewhere [31]. Briefly, samples of ~300 mg dry weight were decarbonated with HCl (37%). After equilibration for 24 h and pH adjustment to neutral values (using ultrapure water), the residue was dried (50°C in an oven) for 72 h and then analyzed by IRMS (MAT 253). Ratios of the heavy over the light stable isotope of nitrogen (δ^15^N) and organic carbon (δ^13^C) were reported in the standard per mil notation (‰) using three certified standards (USGS41, IAEA-600, and USGS40), with an analytical precision of 0.1‰. In parallel to stable isotope analysis, the content of total organic carbon (TOC) and total nitrogen (TN) were determined using an elemental analyzer (HT Flash, Thermo Fisher Scientific) and reported as percentage (%) relative to dry weight.

### Lipid biomarkers extraction, fractionation, and analysis

Lipids from 13–26 g of dry weight samples were extracted with organic solvents (dichloromethane and methanol, 3:1 v:v) using an ultrasound bath (details in [7]). A mixture of internal standards (tetracosane-D_50_, myristic acid-D_27_, and 2-hexadecanol) was added prior to the extraction for quantification (recovery of 78 ± 14 %). The total lipid extract was separated into three polarity fractions according to protocols described elsewhere [7]: non-polar, containing hydrocarbons; acidic, containing fatty acids; and polar, containing alcohols. For analysis, compounds of the acidic fraction were methylated with BF3 (Sigma Aldrich) in MeOH at 80 °C for 30 min. Compounds of the polar fraction were derivatized using BSTFA (Sigma Aldrich) at 80 °C for 60 min. Thus, acids and alcohols were detected as fatty acid methyl esters (FAME) and trimethylsilyl (TMS) derivates, respectively. The hydrocarbons of the non-polar fraction did not need to be derivatized for analysis. Procedural blanks were performed throughout the entire process to confirm that the compounds identified were indigenous to the samples.

Organic compounds in the samples were identified and quantified using a gas chromatography system (6850 GC) coupled to a mass spectrometer (5975 VL MSD) with a triple-axis detector (Agilent Technologies, Santa Clara, CA, USA). The GC-MS operated at an electron ionization of 70 eV and scanned from 50 to 650 m/z (analytical details in [32]). Compound identification was based on retention time and mass spectra comparison with reference materials and the NIST mass spectral database. Quantification was performed with the use of external standards of *n*-alkanes (C_10_ to C_40_), FAMEs (C_6_ to C_24_), and *n*-alkanols (C_14_, C_18_, and C_22_) (Sigma-Aldrich).

### DNA extraction

Metagenomic DNA of the six sediment samples was extracted with E.Z.N.A. soil DNA kit (Omega Bio-Tek, GA, USA) following manufacturer’s instructions. To increase DNA concentration of each sample, separated extractions (from two up to nine) of 0.5 g of dry weight sample were performed and combined at the elution step. The variation in the number of DNA extractions per sample was to achieve a minimum of 5 ng of total DNA in each sample. A negative control of the kit was also conducted following the same procedure without sample. DNA concentrations were determined with Qubit dsDNA HS Assay Kit (ThermoFisher Scientific, MA, USA) using Qubit 3.0 fluorometer (ThermoFisher Scientific). The total amount of DNA obtained was 291 ng for sample A, 6 ng for sample B, 159 ng for sample C, 154 ng for sample D, 20 ng for sample E, and 165 ng for sample F. Genomic DNA extractions were stored at −20°C until sequencing analysis.

### Metagenomic sequencing

Microbial community structure of each sediment sample was determined by the construction of 150 bp paired-end libraries on Illumina NextSeq 550 sequencer (Illumina Inc., San Diego, CA, USA) at the Genomic Service in Madrid Science Park Foundation (Spain). Briefly, DNA was purified, quantified, and quality checked using both Quant-iT PicoGreen dsDNA Assay Kit (Invitrogen, Thermo Fisher Scientific, Waltham, MA, USA) and 2100 Bioanalyzer (Agilent Technologies, Santa Clara, CA, USA). Then, 5 ng (for samples B and E) or 25 ng (for samples A, C, D, and F) were mechanically fragmented on a Bioruptor Sonicator (Diagenode, Liège, Belgium) and DNA fragments were used as an input for library preparation using NEBNext Ultra II DNA Library Prep Kit for Illumina (New England BioLabs, Ipswich, MA, USA). DNA concentration of the negative control of the extraction was below the detection limit of the bioanalyzer and was then excluded from library preparation. Libraries were validated and quantified using the 2100 Bioanalyzer and were then pooled in equimolar concentrations, purified using Agencourt AMPure XP Beads (Beckman Coulter, Brea, CA, USA), and titrated by quantitative PCR using Kapa-SYBR FAST qPCR kit (Kapa Biosystems, Wilmington, MA, USA) in LightCycler 480 System (Roche Molecular Systems, Pleasanton, CA, USA). Final library pools were sequenced using NextSeq 500 High Output kit v2.5 (Illumina) on an Illumina NextSeq 550 sequencer.

### Metagenomic assembly and annotation

Reads from NextSeq sequencing of metagenomic DNA were trimmed and quality-filtered using BBduck (https://sourceforge.net/projects/bbmap) and Sickle (https://github.com/najoshi/sickle). High-quality reads were assembled using MetaSPAdes [33] and open reading frames (ORFs) from scaffolds of at least 1 kb length were predicted using Prodigal in “meta” mode [34]. Taxonomic annotation of predicted genes were performed using DIAMOND blast [35] against UniRef100 database (December 2017) [36], which contains the taxonomic and functional information of the protein sequences. Sequencing coverage of each scaffold was calculated by mapping sample reads using Bowtie 2 [37].

### Metagenomic binning: reconstruction of metagenome-assembled genomes (MAGs)

Scaffolds were clustered into genome bins using different algorithms on the basis of (i) tetranucleotide frequencies and differential coverage across samples using ABAWACA 1.07 [38], (ii) tetranucleotide frequencies using emergent self-organizing maps (tetra-ESOM) [39], and (iii) differential coverage and tetranucleotide frequency patterns using MaxBin 2 [40], using 107 and 40 single-copy marker genes. For ABAWACA analyses, scaffolds were fragmented in two group sizes: 3–5 kb and 5–10 kb. ESOM analyses were only done with the 3–5-kb set. Genome bins resulted from all binning algorithms were aggregated using DAS Tool [41] to obtain non-redundant and high-quality bins and were then manually curated using uBin [42] according to % GC content, coverage pattern, and taxonomic annotation of scaffolds. Final bins were checked for >60% completeness and <10% contamination based on the presence and number of bacterial/archaeal single copy genes (SCGs) [43] using uBin [42]. Additional assessment of the quality of the metagenome-assembled genomes (MAGs) were performed with CheckM v1 [44]. Recovered genomes were dereplicated using dRep [45] to identify groups of highly similar genomes and selected the most complete and representative across genome sets at 99% similarity between genome alignments (strain threshold).

### In situ genome replication indices

To predict if bacteria were actively growing in the samples, we calculated the replication index (iRep index) in the draft-quality MAGs [46]. The iRep index assumes that microorganisms that are actively replicating have higher copies of the genome at the origin of replication than at the terminus. Therefore, iRep values are the ratio between the gene coverage at the origin and terminus of replication, with ≤ 1 indicating the absence of replication. Archaea can have multiple origins of replication, making the iRep assumption invalid, and thus the iRep index was only calculated for Bacteria. iRep indices were calculated for each representative genome with >70% completeness and <10% contamination, based on mapping data generated with Bowtie 2 in “sensitive” mode [37].

### Prokaryotic community profile

Prokaryotic community composition and relative abundance across all samples was calculated by using the abundance of scaffolds containing the gene encoding for the ribosomal protein S3 (*rpS3*). For taxonomic annotation of the *rpS3* gene, scaffolds were searched against the Genome Taxonomy Database (GTDB; release r95) [47] using usearch [48]. Thresholds of percent identity was used for taxonomic classification of the *rpS3* gene. Criteria for *rpS3* gene classification was based on cutoffs similar to those for 16S rRNA gene [2] and were ≥99% for species, 95–98% for genus, 88–94% for family, 84–87% for order, 80–83% for class, 60–79% for phylum, and <60% for kingdom level. Relative abundance of a specific family was estimated by the sum of coverage of the *rpS3*-bearing scaffolds belonging to that family (Table S2). For sample comparison, coverages were normalized to the sequencing depth.

### Metabolic potential of the entire prokaryotic community and individual MAGs

Proteins previously predicted with Prodigal [34], from both the prokaryotic community assemblies and the reconstructed MAGs, were used to assess the metabolic potential of the microorganisms from the Laguna Lejía terrace. The metabolic potential was determined by assigning the protein sequences of the prokaryotic community or the reconstructed MAGs to KEGG (Kyoto Encyclopedia of Genes and Genomes) Orthologs (KO) by homology search using hidden Markov models (HMMs) and score thresholds for reliable KO assignments [49]. Finally, a list of key enzymes, KO numbers, and genes from specific metabolic pathways of interest in this extreme environment (e.g., arsenic metabolism or resistance) were used in this study (Table S3). For the prokaryotic community, the relative abundance of a metabolic pathway was estimated by the sum of scaffold coverage bearing the gene(s) of interest (Table S3). Scaffolds bearing genes of interest were previously normalized to the sequencing depth. For the MAGs, the presence or absence of a given metabolic pathway is shown based on the presence of at least one specific gene to that pathway.

To verify that *amo* genes identified in archaeal and bacterial MAGs were not contamination from binning, gene arrangement, and phylogenetic analyses were performed with *amo* genes from the Lejía MAGs and those from the archaeal and bacterial genomes present in reference databases (Text S1). In addition, to check for the presence of carbon monoxide dehydrogenases (CODH) form I or II in the Lejía MAGs, a phylogenetic analysis was performed with CoxL sequences from the Lejía genomes and those from the Swiss-Prot database (Text S2).

### Phylogenomics

Taxonomic classification of recovered MAGs was conducted with GTDB-tk [47, 50] against the GTDB (release r95) [51]. The novelty of MAGs at a specific taxonomic level was considered when they were annotated as unclassified [52].

Using the GTDB-Tk assignments above, the 591 MAGs were split into Bacteria (572 genomes) and Archaea (19 genomes). Protein sequences were predicted with Pyrodigal v2.0.4 [34, 53] and used to create a local database for each domain. We used HMMER v3.3.2 [54] to search against each domain database, using the profiles of 30 universal ribosomal proteins from PhyloSift [55] and an e-value cutoff of 1E−10. Sequences were extracted with Pullseq (https://github.com/bcthomas/pullseq) and all cases of multiple homologs in a genome were removed. The datasets were aligned with MAFFT E-INS-I (v.7.505) [56], trimmed with BMGE version 1.12 [57] (BLOSUM30), and concatenated. For each concatenation, we constructed phylogenies in IQ-TREE v2.0.5 [58] with the model automatically selected by Modelfinder [59] out of the WAG, JTT, and LG replacement matrices (-m MFP -mset WAG,JTT,LG). For Archaea, we also constructed a phylogeny under the posterior mean site frequency (PMSF) model [60] LG+C60+R10, using the previous phylogenies as guide trees. Branch supports were calculated with 1000 ultrafast bootstrap [61] and 1000 aLRT SH-like [62] replicates. Phylogenies were visualized with iTOL v5 [63].

### Statistical analysis

A Pearson correlation analysis was performed in R software [64] with the package “rstatix” (v.0.7.2) [65] between the abundance of each phylum and the concentration of soluble elements and minerals to elucidate the variables that may have contributed most to explain the variability of the microbial communities in the terrace profile. Then, geochemical and mineral variables with statistically significant correlations (*p*-values <0.05) above 0.8 and below −0.8 were included in a principal component analysis (PCA). The PCA was performed with the software CANOCO5 v.5.12 (Microcomputer Power, Ithaca, NY) to explore the variability in the prokaryotic composition at the phylum level between the six terrace layers of the Laguna Lejía. Then, the environmental variables were projected in the ordination space to aid interpretation. The abundance of phyla and environmental variables were centered prior to the statistical analysis. A second analysis based on principal coordinate analysis (PCoA) using the Bray–Curtis distances at the *rpS3* gene level was performed to confirm the differences in the prokaryotic community composition between the six sediment layers of the Laguna Lejía terrace. Prokaryotic community richness and Shannon–Wiener diversity index (H’) were calculated using the vegan package in R [66].

## Results

### Age, mineralogy, and elements of the *Laguna* Lejía terrace

The organic carbon of samples A (0–7 cm), C (9–35 cm), and F (70–95 cm) (Fig. 1f and g) were radiocarbon dated to 20,920 ± 70 years BP (before present), 22,180 ± 70 years BP, and 21,580 ± 70 years BP, respectively. Considering a reservoir effect (discussed later), ^14^C-corrected ages were estimated in ~11,000 years BP for sample A, ~10,650 years BP for sample C, and ~10,300 years BP for sample F (Figure S1).

**Fig. 2:**
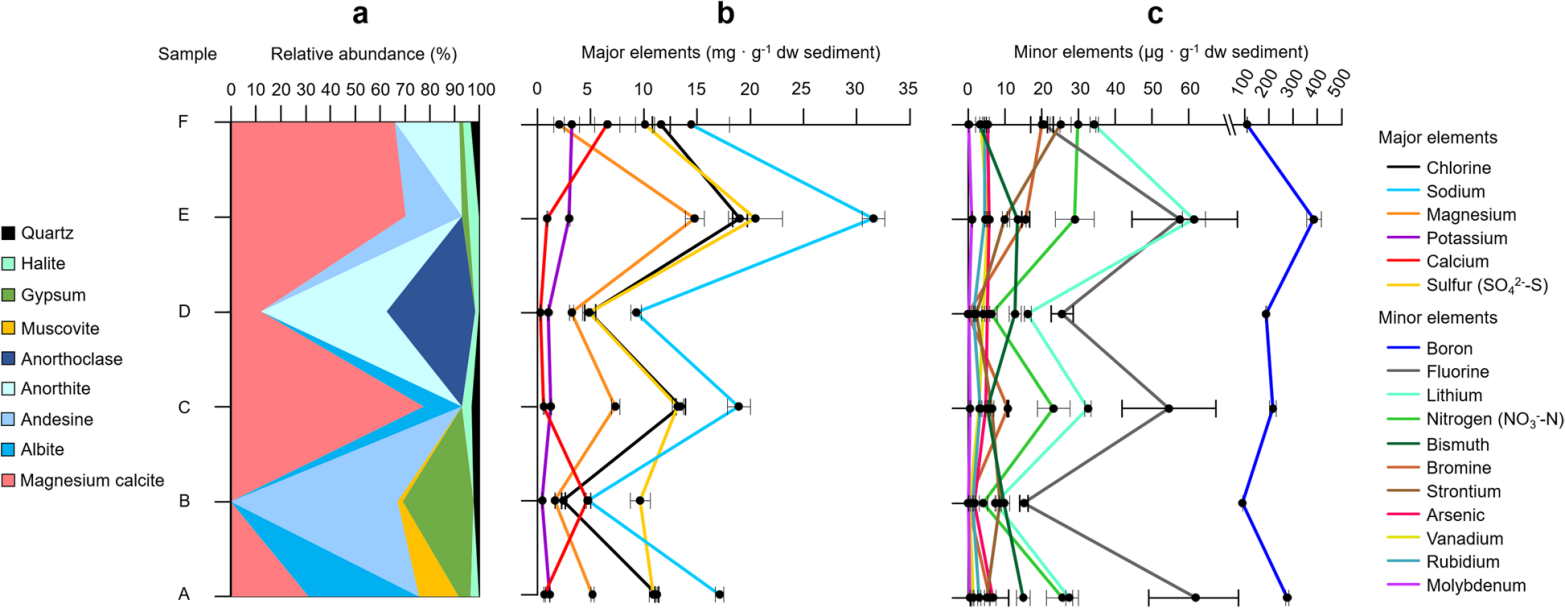
Mineralogical and geochemical composition of the six sediment samples from the Laguna Lejía terrace (10.3–11 kyr BP). **a** Mineralogical composition of the crystalline fraction measured by X-ray diffraction (XRD). The minerals with the blue color scale are feldspars. **b** Major and **c** minor elements in the soluble fraction of sediment samples. The sulfur concentration was calculated from sulfate, and the nitrogen concentration was calculated from nitrate since nitrite was below the quantification level (1.6 ppb). Error bars represent the standard deviation of triplicate measurements

The mineral composition of the terrace sediments showed differences along the vertical profile (Fig. 2a). Samples A, C, E, and F were composed mainly of magnesium calcite (31–78%), a variable proportion of feldspars (anorthite, andesine, and albite) (16–45%), and halite (3–4%). By contrast, samples B and D were mainly composed of feldspars (67–86%) and, in the case of B, gypsum (29%).

The concentration of elements in the soluble fraction of the sediment samples showed a similar trend as the mineralogy (Fig. 2b and c). The samples with the highest proportion of magnesium calcite and halite (A, C, E, and F) showed the highest concentrations of elements in general, especially Na (14–32 mg · g^-1^ dw), Cl (11–19 mg · g^-1^ dw), Mg (2–15 mg · g^-1^ dw), and SO_4_^2-^-S (5–20 mg · g^-1^ dw). By contrast, the samples with the highest proportion of feldspars and/or gypsum (B and D) showed the lowest concentration of elements, with the exception of SO_4_^2-^-S in B (10–13 mg · g^-1^ dw). Other relevant elements in the terrace, although present in minor concentrations, were NO_3_^-^-N (4–30 µg · g^-1^ dw), Li (7–61 µg · g^-1^ dw), or Ar (2–7 µg · g^-1^ dw). The pH of the six sediment samples was similar (pH = 8.2 ± 0.1).

### Bulk geochemistry and lipids of the *Laguna* Lejía terrace

**Fig. 3:**
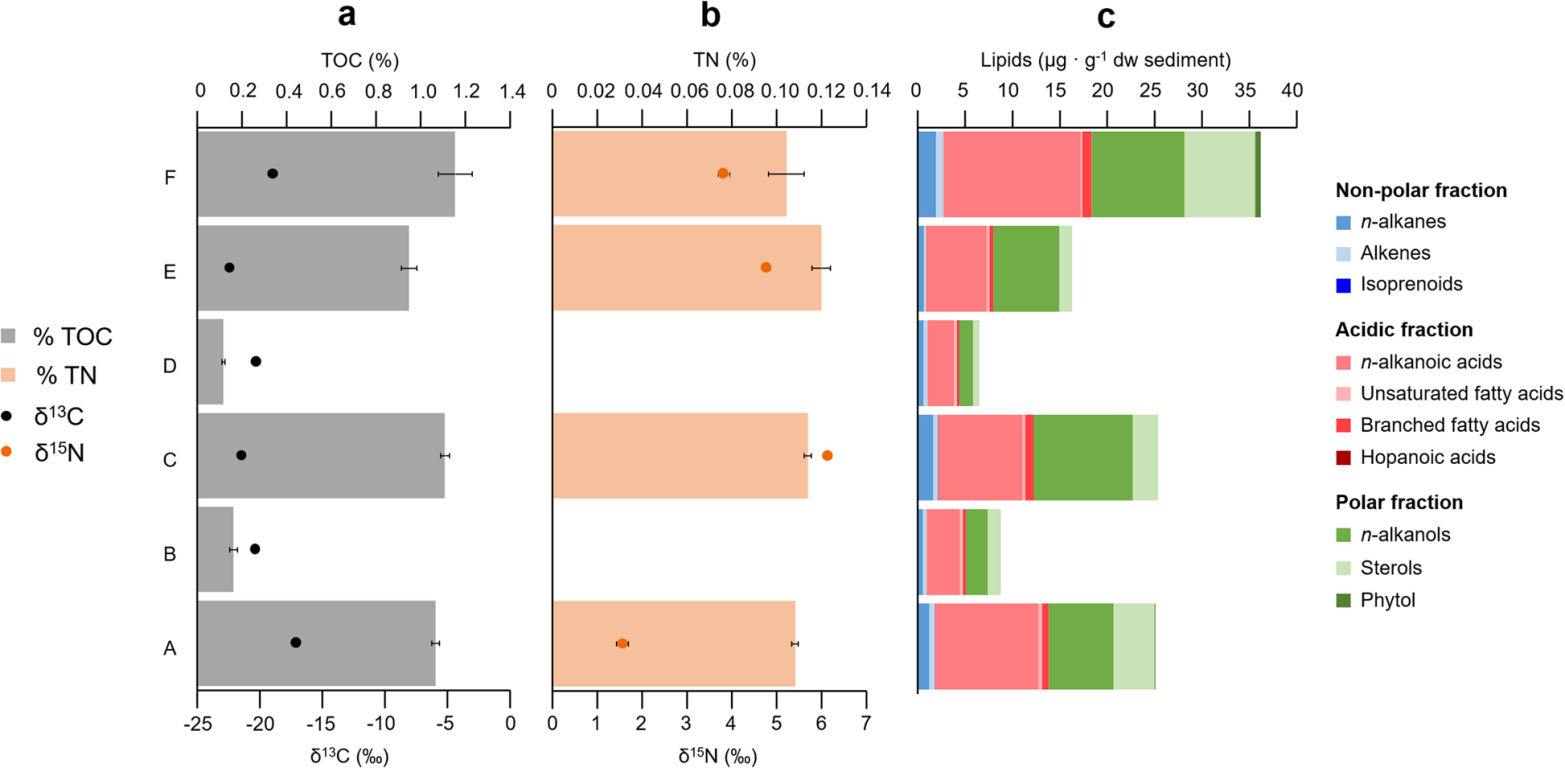
Lipid and bulk isotopic composition of the organic matter in the six sediment samples from the Laguna Lejía terrace. **a** Total organic carbon (TOC) and stable-carbon isotopic ratio (δ^13^C). **b** Total nitrogen (TN) and stable-nitrogen isotopic ratio (δ^15^N). TN concentration was not detected in samples B and D. **c** Composition of the three polarity fractions of lipids (non-polar, blue color shades; acidic, red color shades; and polar, green color shades). Error bars represent the standard deviation of triplicates. Error bars in δ^13^C are behind the dots and are smaller than the dots’ size

The concentration of TOC, TN, and total lipids followed a similar trend to that of minerals and elements (Fig. 3a, b, and c). Samples A, C, E, and F contained the highest concentrations of TOC (0.9–1.2 %), TN (0.10–0.12 %), and total lipids (16–36 µg · g^-1^ dw), while samples B and D showed the lowest TOC (0.1–0.2 %), total lipids (7–9 µg · g^-1^ dw), and TN (not detected). The δ^13^C of TOC varied along the terrace profile from −17‰ to −22‰, and the δ^15^N of the TN, from 1.6‰ to 6.1‰.

The most abundant lipid compounds were those from the acidic and polar fractions, particularly the straight-chain or *normal* alkanoic acids (a.k.a. *n*-fatty acids) (3–14 µg · g^-1^ dw) and *n*-alkanols (1–10 µg · g^-1^ dw), as well as sterols (1–7 µg · g^-1^ dw). By contrast, the *n*-alkanes, alkenes, and isoprenoids (non-polar fraction) were detected in lower concentration in all samples (<2 µg · g^-1^ dw). The molecular distributions of *n*-alkanes (C_16_-C_29_), *n*-fatty acids (C_12_-C_30_), and *n*-alkanols (C_14_-C_28_) showed compounds of both low (≤C_21_) and high (>C_21_) molecular weight (Figure S2, S3, and S4). In the* n*-alkanes distribution, a unimodal pattern was found with dominance of C_21_ or C_27_ depending on the sample (Figure S2). In the *n*-fatty acids series, a bimodal distribution was defined by C_16_ and C_24_ (Figure S3). The *n*-alkanols series was dominated by C_22_, except in the sample A, where C_24_ was more abundant (Figure S4). Other lipid compounds detected in concentrations relevant in the three polarity fractions were the alkenes C_18:1_ and C_22:1_ (Figure S2), a few branched (mostly *i/a*-C_15:0_) and monounsaturated (mostly C_18:1_) fatty acids (Figure S3), and sterols mainly consisting of phytosterols (β-sitosterol, stigmasterol, and campesterol) and cholesterol or cholesterol-derived compounds (Figure S4).

### Prokaryotic community structure and influence of environmental conditions

**Fig. 4:**
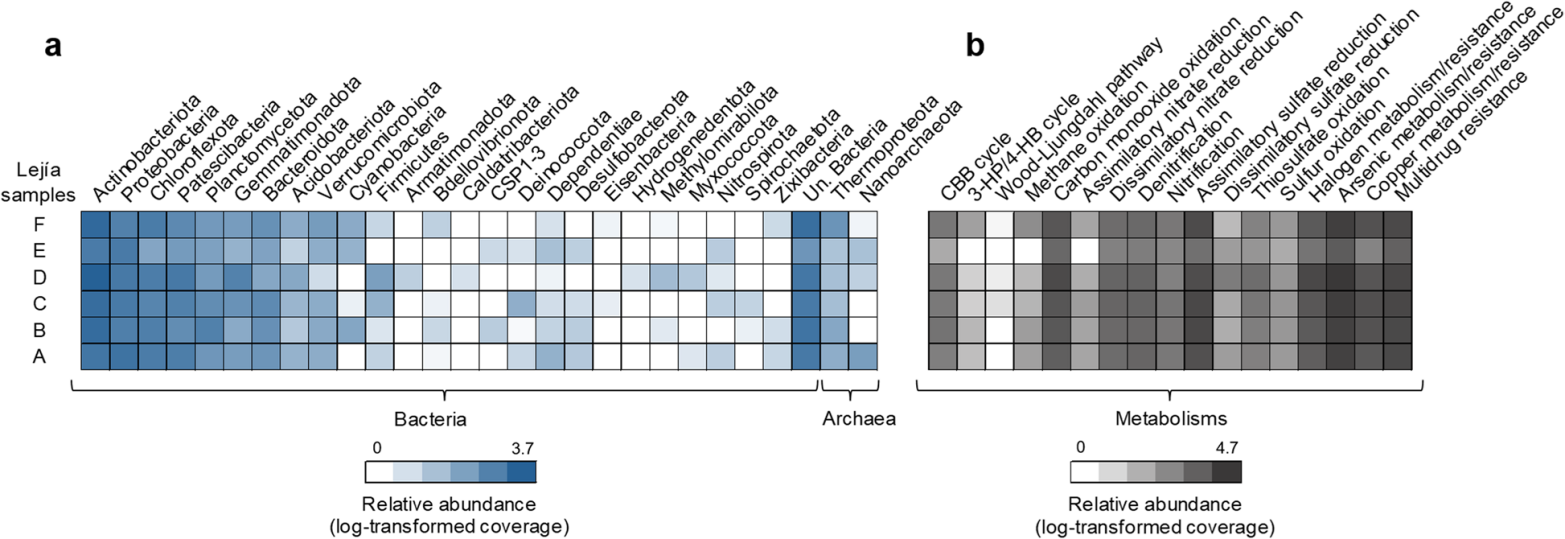
Prokaryotic community composition and metabolic potential of the Laguna Lejía terrace sediments (A to F). **a** Prokaryotic community composition at the phylum level (blue color scale) based on the *rpS3* gene similarity to the GTDB database (r95). The heatmap is made by summing scaffold coverage containing the *rpS3* gene, normalized by sequencing depth, summed by phylum and sample, and then log-transformed to help visualization of the less abundant phyla. **b** Prokaryotic metabolic potential (gray color scale) based on the prediction of key enzymes. The heatmap is made by summing scaffold coverage containing genes encoding for specific key enzymes, normalized by sequencing depth, summed by metabolism and sample, and then log-transformed to help visualization of the less abundant metabolisms. Key genes and enzymes involved in each specific metabolism are listed in Table S3. No enzymes were annotated to the 3-HP bi-cycle and N_2_ fixation. "Un." means unclassified

We used the taxonomic annotation of the *rpS3* gene from assembled metagenomes reads against GTDB to identify the prokaryotic community members and their relative abundance on the Laguna Lejía terrace. The most abundant bacterial phyla in the six samples were Actinobacteriota (23–40%), followed by Proteobacteria (12–35%), Chloroflexota (4–16%), Patescibacteria (also known as candidate phylum radiation or CPR) (7–15%), Planctomycetota (4–13%), Gemmatimonadota (2–9%), and Bacteroidota (1–8%) (Fig. 4a). Other bacterial phyla were below 3% in the six samples. The six samples also showed a relatively high proportion of unclassified bacteria at the phylum level (8–23%). Below the phylum level, the orders Gaiellales, Solirubrobacterales, and UBA5794 (Actinobacteriota), as well as the class Gammaproteobacteria and the order Kilonellales (Proteobacteria) were the most abundant taxa in the Lejía terrace (>5% in at least one sample) (Table S2). Only two archaeal phyla were identified, Thermoproteota and Nanoarchaeota (the latter, DPANN superphylum). The phylum Thermoproteota was mainly present in samples B (2%) and F (5%), and Nanoarchaeota, in the sample A (3%). The six samples showed Shannon indices (H’) that vary from 3.9 to 4.8 and a richness (S) that vary from 175 to 334 (Figure S5).

The PCA to explore the variability in the prokaryotic community composition at the phylum level between sediment layers showed three different groups of samples (A, C, and E; B and F; and D) (Figure S6). The PCoA analysis at the *rpS3* gene level also confirmed the prokaryotic community differences, with sample D being the most different (Figure S7). Despite such differences, the distinct grouping of samples based on geochemistry and mineralogy (A, C, E, and F versus B and D, Figs. 2 and 3) indicated a low relationship between the prokaryotic community structure and the geochemistry and mineralogy of the Laguna Lejía terrace. Still, significant Pearson correlations (*p*-value < 0.05) were identified between certain phyla and the geochemistry and/or mineralogy (Table S4 and S5). For instance, the highly abundant Actinobacteriota, mainly comprised by the orders Gaiellales, Solirubrobacterales, and UBA5794, were positively correlated (*p*-value < 0.05) with anorthoclase and anorthite feldspars.

### Predicted prokaryotic metabolisms in the *Laguna* Lejía terrace

The metabolic potential of the prokaryotic community from the Laguna Lejía terrace was determined based on the relative abundance of predicted and annotated key enzymes (Table S3) in metagenome assemblies (Fig. 4b and Table S6). The most relevant metabolisms predicted in the Lejía prokaryotes were those related to carbon, nitrogen, and sulfur biogeochemical cycles as well as to the transformation of toxic compounds (e.g*.*, arsenic and halogenated compounds).

Predicted enzymes involved in assimilatory sulfate reduction (e.g., CysN and CysJ) comprised 10–17% of the total predicted and annotated proteins, and those for thiosulfate oxidation (e.g., SoxA, SoxB, or SoxC), 1–3%. A minor proportion was detected for enzymes involved in dissimilatory sulfate reduction (e.g*.*, Sat, AprA, AprB, DrsA, or DsrB) (0.1–1.3%). Predicted enzymes involved in dissimilatory nitrate reduction (e.g., NarG, NapA, or NrfA) comprised 3–5%, and denitrification enzymes (e.g., NirS, NirK, NorB, or NosZ) accounted for 4–6%. In addition, predicted enzymes involved in nitrification and/or methane oxidation (*e*.g., AmoA/PmoA) comprised 1–3%, and those involved in carbon monoxide (CO) oxidation (e.g*.*, CoxL, CoxM, or CoxS) were present in high relative abundance (7–15%).

The most relevant carbon fixation pathway predicted in the prokaryotic community was the Calvin–Benson–Bassham (CBB) cycle (1–3%), based on the annotation of Rubisco enzymes (RbcL, RbcS) and a phosphoribulokinase (prkB) involved in CO_2_ assimilation. Other enzymes specific for the Wood–Ljungdahl (WL) pathway (e.g*.*, anaerobic CO dehydrogenase) and the 3-hydroxypropionate/4-hydroxybutyrate (3-HP/4HB) cycle (e.g*.*, 3-hydroxypropionyl-CoA dehydratase) were also predicted in low abundance (<0.5%).

Interestingly, the prokaryotic community of the Laguna Lejía terrace showed high relative abundance of predicted enzymes involved in arsenate reduction (e.g., ArsA, ArsB, ArsC, ArsH, or Acr3) and arsenite oxidation (e.g., AoxA or AoxB) (23–27%), as well as in the transformation of halogenated compounds (e.g*.*, DehH or DhaA) (7–14%), and multidrug efflux pumps (15–20%) in the six sediment samples.

### Diversity and novelty of MAGs recovered from the *Laguna* Lejía terrace

**Fig. 5:**
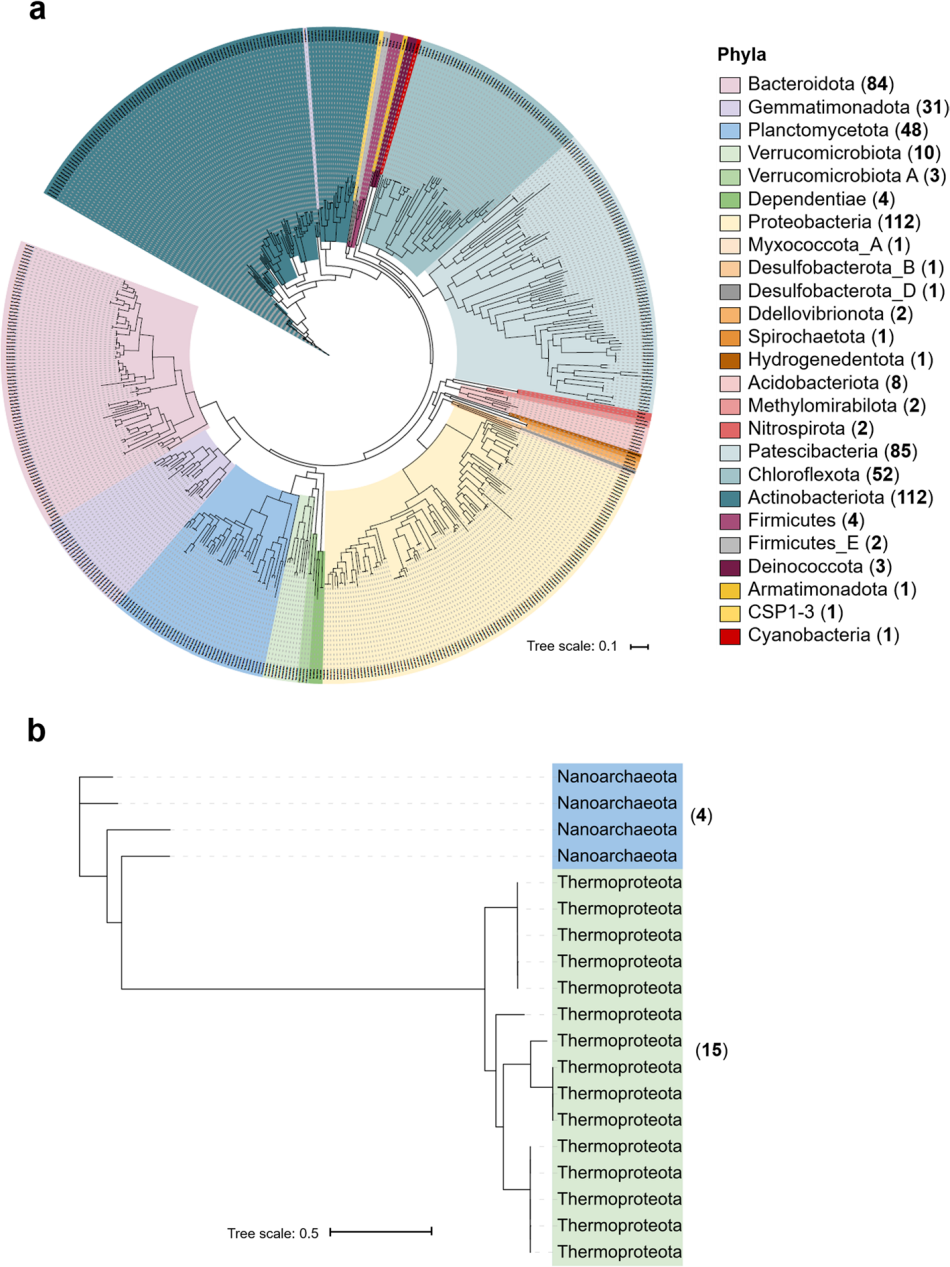
Maximum Likelihood unrooted phylogenies (model selected in IQ-TREE by Modelfinder) of the new **a** 572 bacterial genomes and **b** 19 archaeal genomes reconstructed from the Laguna Lejía terrace. Phylogenies were constructed using a concatenation of 30 ribosomal proteins. In brackets, the number of MAGs at the phylum level

A compendium of 591 metagenome-assembled genomes (MAGs) were recovered from the Laguna Lejía terrace, 572 being from bacteria and 19 from archaea. Bacterial MAGs belonged to 22 phyla, mainly to Actinobacteriota (112), Proteobacteria (112), Patescibacteria (85), Bacteroidota (84), Chloroflexota (52), and Planctomycetota (48) (Fig. 5a). In contrast to the broad variety of bacterial phyla in the Laguna Lejía terrace, archaeal MAGs only belonged to two phyla, Thermoproteota (15) and Nanoarchaeota (4) (Fig. 5b).

**Fig. 6:**
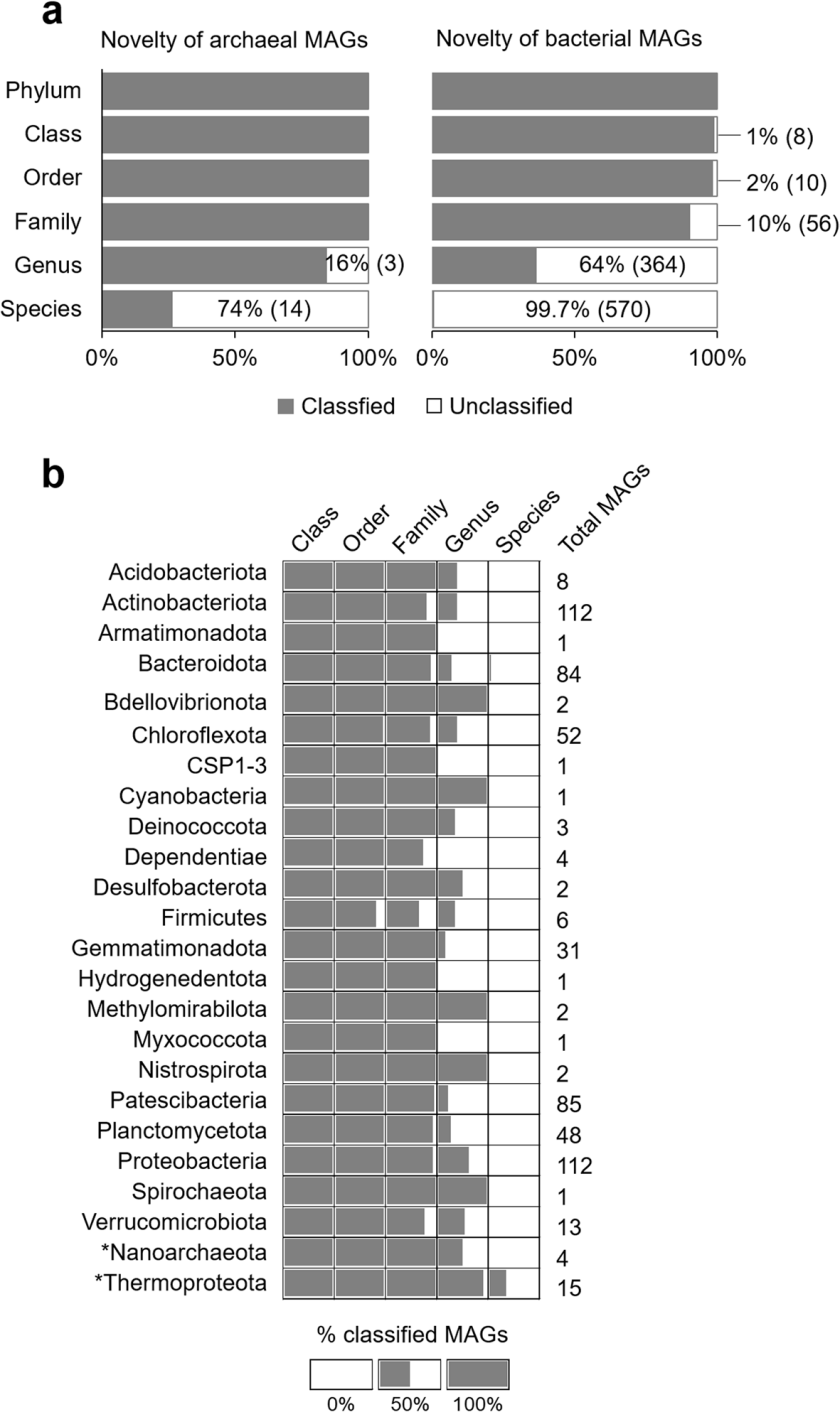
Novelty quantification of total bacterial and archaeal MAGs from the Laguna Lejía terrace. **a** Proportion of classified (gray) and unclassified (white) total archaeal and bacterial MAGs at different taxonomic levels using GTDB. In brackets, the number of unclassified MAGs. **b** Proportion of classified (gray) and unclassified (white) total archaeal (asterisks) and bacterial MAGs grouped by phylum at different taxonomic levels.

All bacterial MAGs were assigned to a known phylum, but some of them were unclassified at lower taxonomic levels (Fig. 6a). Specifically, 1% of the MAGs were not assigned to a known class, 2% to a known order, 10% to a known family, 64% to a known genus, and 99.7% to a known species. The MAGs that were not assigned to a known class or order were from phyla Actinobacteriota (4%, 4 MAGs), Patescibacteria (5%, 4 MAGs), Firmicutes (17%, 1 MAG), and Chloroflexota (2%, 1 MAG) (Fig. 6b). All archaeal MAGs were assigned up to a known family (Fig. 6a). Below the family level, about 16% and 74% of the archaeal MAGs were not assigned to a known genus and species, respectively.

### Metabolic potential and replication rates of MAGs from the *Laguna* Lejía terrace

**Fig. 7:**
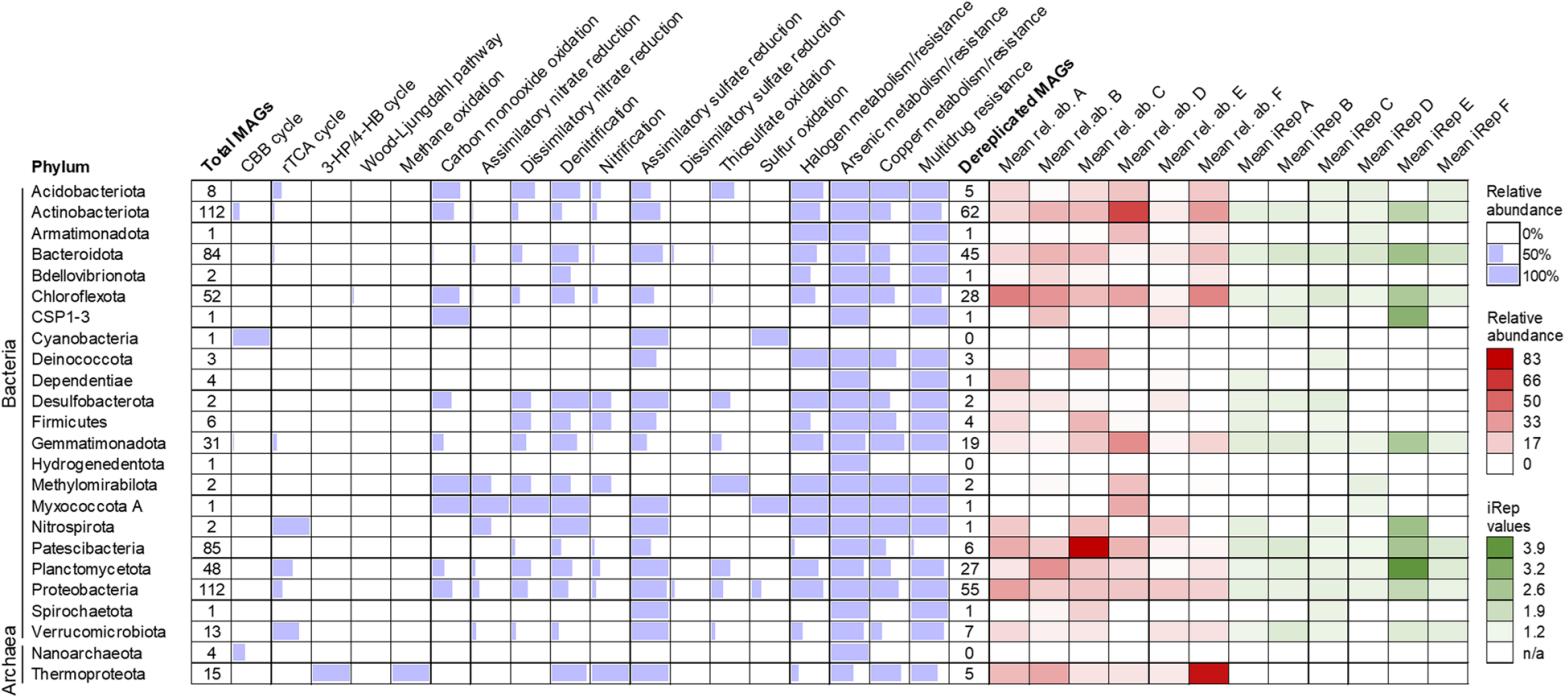
Genomic features, predicted metabolic potential, and replication (iRep) indices of 591 MAGs recovered from the Laguna Lejía terrace grouped by phylum. The completeness of purple bars within cells represents the abundance of total MAGs that have annotated at least one key enzyme for a specific metabolic pathway within a phylum. Key enzymes involved in each specific metabolism are listed in Table S3. No enzymes were annotated to the 3-HP bicycle and N_2_ fixation. Color gradients represent the mean relative abundances (red) or iRep values (green) of dereplicated MAGs for each phylum. The genomic features and predicted metabolic potential and replication indices of each individual MAG are shown in Table S7

The predicted metabolic potential of the 591 prokaryotic MAGs, their relative abundances along the Lejía terrace profile, and their replication indices (calculated only on those that are unique over the 591, i.e., dereplicated) are described in Table S7. A summary of the metabolic potential, relative abundances, and replication indices of the MAGs grouped by phylum is shown in Fig. 7.

CO oxidation, dissimilatory nitrate reduction, denitrification, assimilatory sulfate reduction, and thiosulfate oxidation were the most abundant and widespread metabolisms related to carbon, nitrogen, and sulfur biogeochemical cycles in the recovered MAGs (Fig. 7). By contrast, nitrification, dissimilatory sulfate reduction, and three CO_2_ fixation pathways identified in the Lejía MAGs (CBB, 3-HP/4-HB, and WL) were less abundant.

Potential nitrite oxidoreductases, involved in nitrification, were annotated in Actinobacteriota, Proteobacteria, Bacteroidota, and Firmicutes, among other phyla (Table S7). Since the KEGG database does not differentiate between nitrite oxidoreductases and nitrate reductases (e.g., K00370 and K00371), both annotations were considered. Ammonia monooxygenases (also involved in nitrification) were identified in Thermoproteota and one unclassified Actinobacteriota MAG (class UBA4738, order AC-67; Table S7). Gene arrangement analysis (Figure S8) and phylogenetic trees (File S1 and S2) of *amoABC* genes confirmed their presence in the archaeal MAGs and in the bacterium MAG. For instance, *amo* gene arrangements were similar between closely related genomes, with few changes in orientation (Figure S8). In addition, the bacterial *amo*C gene from the Actinobacteriota MAG was located within the bacterial *amo*C genes in the phylogenetic tree (File S1), ruling out possible contamination of archaeal *amo* genes during binning.

Enzymes involved in dissimilatory sulfate reduction, such as adenylylsulfate reductases, were predicted in Bacteroidota and α- and γ-Proteobacteria (Table S7). Apart from Cyanobacteria and some Actinobacteriota MAGs, the Rubisco (rbcL or rbcS) and/or phosphoribulokinases involved in CBB were predicted in γ-Proteobacteria, Gemmatimonadota (order SG8-23), Plantomycetota (Phycisphaerales), and one Nanoarchaeota MAG (Pacearchaeales) (Table S7). Moreover, CO dehydrogenases form I, involved in CO oxidation, were also predicted in a large proportion of the Lejía MAGs (Figure S9). Interestingly, most of the MAGs from the Laguna Lejía terrace encoded enzymes for arsenic (e.g., Ars and Aox) and copper (e.g., Cop and Cus) metabolism and/or resistance, transformation of halogenated compounds (DehH or DhaA), and multidrug efflux systems.

**Fig. 8:**
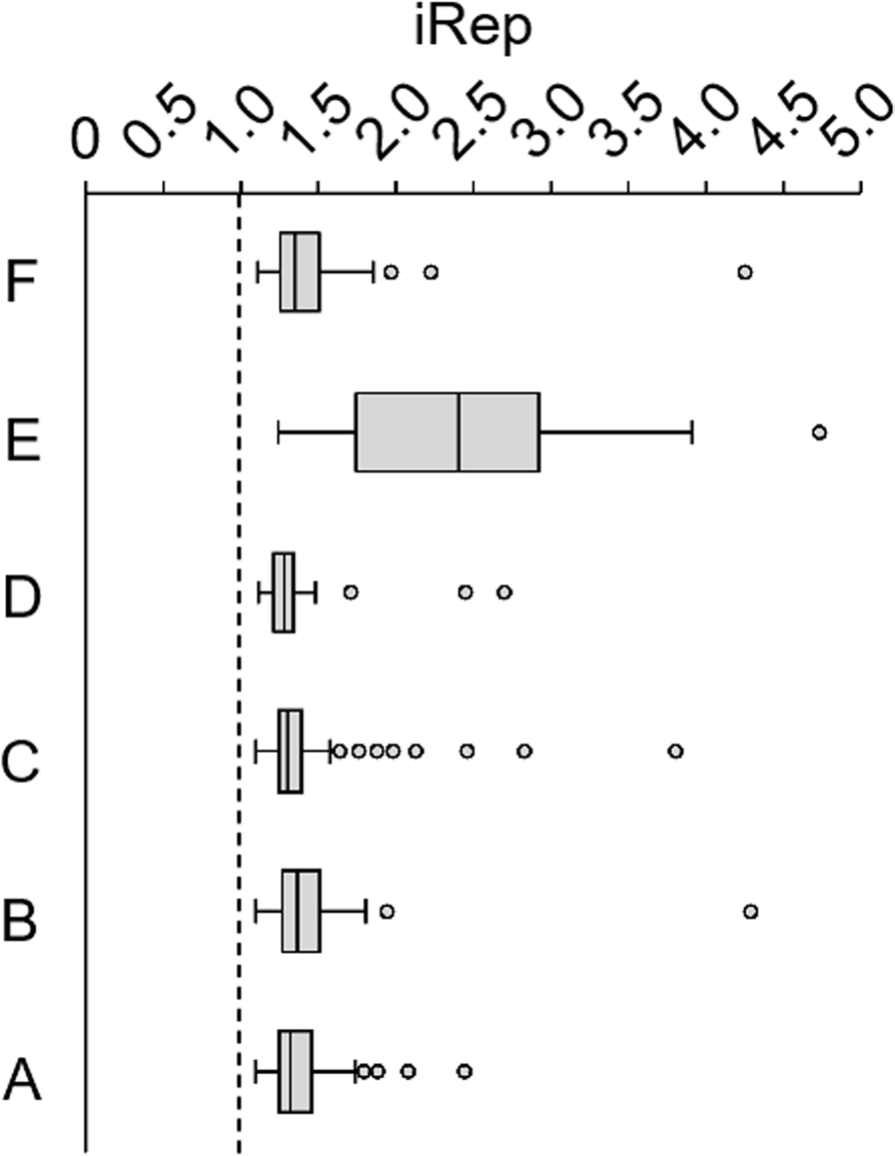
Predicted genome replication (iRep values) of bacterial MAGs from the Laguna Lejía terrace at the time of sampling. The dashed line represents the threshold above which population genomes are considered to be replicating. N in each sample is: 73 for A, 73 for B, 111 for C, 67 for D, 41 for E, and 73 for F

Average genome replication (iRep) values of bacterial MAGs were between 1.3 and 1.4 in samples A–D and F, and 2.4 in sample E (Fig. 8). In all samples, iRep values showed a wide variability, ranging from ~1.1 to 4.3 in samples B and F and up to 4.7 in sample E.

## Discussion

### Sediments from the *Laguna* Lejía terrace revealed a saline, sulfur-rich environment with large-scale water level fluctuations between ca. 10.3 and 11 kyr BP

The geochemistry, mineralogy, and radiocarbon dating of the Laguna Lejía terrace allowed the reconstruction of the hydrogeochemical history of the lake over the last millennia. Conventional ^14^C ages (i.e., raw ages) of the sedimentary organic matter were similar along the 1-m high terrace profile, with the middle layer (C; ~22,000 years BP) being older than the upper (F; ~21,500 years BP), and the lower (A; ~21,000 years). These relatively high radiocarbon ages and their inversion with depth suggest a reservoir effect that could overestimate the actual age of the sediments. The reservoir effect is the phenomenon whereby the radiocarbon ages of lake sediments are anomalously old because there is an input of dissolved ancient carbonates from groundwater (with a lower ^14^C level than the atmosphere) entering the lake [67, 68]. Conventional radiocarbon ages of another 4-m high terrace from Laguna Lejía were lower (11,700–15,490 years BP) than those of our samples, but showed a similar age inversion with depth [16–18] consistent with changes in the degree of a reservoir effect as lake water level fluctuated [16]. Based on the location of our 1-m high profile relative to the 4-m high of the total terrace [16, 18], our sediment samples correspond to the lithologic units II and III and should have ^14^C-corrected ages between 10,300 and 11,000 years BP (Figure S1). Given this chronostratigraphic relationship, we applied a reservoir effect correction of ~9900–12,300 years, resulting in ^14^C-corrected ages of ~11,000 years BP for sample A (bottom), ~10,650 years BP for sample C (middle), and ~10,300 years BP for sample F (top). If the hypothesis of the reservoir effect is the factor explaining the relative old ages and their inversion with depth, then the section of the terrace studied here correspond to a time interval of ca. 700 years.

The geochemistry of the Laguna Lejía terrace provided evidence for a sulfur-rich saline environment influenced by the volcanic activity and climatic conditions of the region. The Laguna Lejía is located in a volcanic area near the Lascar volcano [14], which is currently active [19] and emits high concentrations of SO_2_, HCl, H_2_S, and HF [69]. The low precipitation (<200 mm year^-1^) and high evaporation rates (1500 mm year^-1^) of the region [15, 17] favor the evaporation of the water and, consequently, the concentration of salts. For instance, the most abundant elements and the pH in the Laguna Lejía terrace are consistent with the lake water chemistry reported in Grosjean et al*.* (1995) [17] (pH of 8.7 and high concentrations of SO_4_^2-^ (28 g L^-1^), Cl^-^ (9.9 g L^-1^), Mg^2+^ (5.9 g L^-1^), and Na^+^ (1.6 g L^-1^)). In addition, metals and metalloid elements detected in different concentrations in the terrace (i.e., Li, B, F, and As) can also be explained as a result of the volcanic activity or weathering of extensive volcanic rocks [70–72].

Differences in the concentration of elements, mineralogy, lipid biomarkers, TOC, and TN along the Lejía terrace profile suggested large-scale water level fluctuations over the ~700 years. Samples A, C, E, and F had a relatively higher proportion of magnesium calcite and halite than B and D (Fig. 2a). This mineralogical composition in A, C, E, and F coincided with a higher concentration of elements (e.g., Cl, Na, SO_4_^2-_^S, and Mg), lipid biomarkers, TOC, and TN than in B and D (Fig. 2b), suggesting periods of desiccation (A, C, and E–F) and water recharge events (B and D). As water evaporates, chemical species (Cl^-^, Na^+^, Mg^+2^, CO_3_^2-^, etc.) and organic matter (i.e., TOC and lipid biomarkers) tend to concentrate in the water column of the lake and accumulate in the sediments. By contrast, lake water recharge dilutes inorganic and organic material in the water column and accumulates relatively less in the sediments. Interestingly, Geyh et al. (1999) [16] and Grosjean et al. (1995) [17] described three large-scale water level fluctuations (25 m to 7 m) in the Laguna Lejía between 10,300 and 11,000 years BP. According to our geochemical and mineralogical results, five periods of alternating desiccation (A, C, and E–F) and water recharge events (B and D) might have occurred in the Laguna Lejía during these ~700 years.

### *Laguna* Lejía terrace preserves aquatic/terrestrial plant remnants and harbors metabolically active microorganisms

The higher proportion of polar and acidic lipid families over the non-polar ones in all sediment layers suggested a well-preserved biomass in the terrace. The *n*-alkanes are the most resistant of the three lipid families to chemical alteration over time [73], whereas *n*-fatty acids and *n*-alkanols contain functional groups (i.e., carboxylic and hydroxyl, respectively) that are more prone to decay. The higher relative abundance of functionalized (*n*-fatty acids and *n*-alkanols) over non-functionalized (*n*-alkanes) hydrocarbons in all sediment layers (Fig. 3c) suggested good preservation of lipids in the Laguna Lejía terrace.

The molecular distribution of the major lipid families (i.e., *n*-alkanes, *n*-fatty acids, *n*-alkanols, and sterols) revealed preservation of ancient biomass from aquatic and terrestrial sources. Despite the taxonomic specificity of lipid biomarkers is limited (i.e., they can only be assigned to a general group of organisms), their stability in the geological record for billions of years [74, 75] makes them relevant biomolecules for paleobiological reconstructions [7, 76]. The identification of lipid biomarkers attributed to aquatic macrophytes (C_21_ *n*-alkane; Figure S2) [77] and terrestrial plants (C_27_ *n*-alkane, C_24_ *n*-fatty acid, and/or C_22_ *n*-alkanols; Figure S3 and S4) [78] suggested the presence of ancient plant biomass inhabiting the lake or its surroundings when the water level was higher. The presence of phytosterols (campesterol, stigmasterol, and β-sitosterol) from mostly vascular plants [79] in all samples also supported this notion and is consistent with the vegetation associated to the Laguna Lejía [80].

A prominent peak at C_16_ within the *n*-fatty acids distribution also hinted toward a contribution from bacteria in the terrace [7, 81]. This bacterial community in the Laguna Lejía terrace was presumably active at the time of collection according to in silico growth predictions of MAGs (average iRep values between 1.3 and 1.4 in samples A–D and F, and 2.4 in sample E) (Figs. 7 and 8). The replication index is calculated based on the DNA sequence coverage at the origin over the terminus of replication [46]. Therefore, iRep values greater than 1 indicate replication of genomes, and values greater than 2 may point out multiple replication forks. Certain bacterial MAGs have values close to 1 (e.g., 1.09 in sample C), suggesting slow replication rates. By contrast, the high iRep values in some of the MAGs, especially in sample E (i.e., up to 4.7 in E) (Fig. 8), may be explained by the relatively high concentration of key nutrients in this sediment layer, such as SO_4_^2-^-S or NO_3_^-^-N, that may promote bacterial growth.

The possibility that a fraction of the microbial biomass (here measured as DNA and lipids) detected in the Lejía terrace was ancient may not be ruled out, as ancient samples tens to billion years old usually contain traces of DNA, proteins, and lipids of microbial origin [7]. Each biomolecule has different preservation rates over time, with that of lipids being the highest (billion years [74]) and DNA the lowest (~1 million year [82]). In addition, the good insulation of biomolecules from UV radiation and high temperatures, as it the case of the Laguna Lejía terrace, also plays a key role in the preservation of DNA and lipids [82].

### Desiccation of the *Laguna* Lejía provided new ecological niche opportunities for microorganisms

The prokaryotic community composition of the Laguna Lejía terrace differed from that reported in the water and wet sediments of the lake in previous studies, suggesting that lake desiccation resulted into a new ecological niche for microorganisms adapted to drier conditions. The bacterial community of the wet sediments showed Bacteroidota, Firmicutes, Spirochaeota, and Proteobacteria as the most abundant phyla [21, 22], while the dried terrace was dominated by Actinobacteriota, Proteobacteria, Chloroflexota, and the Candidate Phylum Patescibacteria (or CPR) (Fig. 4a). Although Actinobacteriota is a common phylum in aquatic and terrestrial habitats worldwide, its dominance in the terrace (23–40%) compared to the wet sediments (~2.5%) [22], and its common presence in soils and rocks of the Atacama Desert [83–85], supports a microbial community shift likely driven by extended periods of drought during water level fluctuations.

In contrast to Actinobacteriota, the high proportion of CPR bacteria found in the Lejía terrace (up to 15%) has not been reported in Atacama sediments or rocks, but detected in smaller relative abundances in Lomas Bayas, María Elena, and Yungay [6], and in the Salar de Llamara [86, 87]. The relatively high proportion of CPR, and the presence of Nanoarchaeota (DPANN superphylum) in the Laguna Lejía terrace suggested extensive microbial interactions, potentially even between bacterial and archaeal communities [88]. The CPR bacteria are a group of ultra-small, genome-reduced bacteria with host-associated lifestyles [89], either parasitic [90] or symbiotic [91], to meet their metabolic requirements [92]. Similarly, Nanoarchaeota are also small-size, genome-reduced archaea that are parasitic or symbiotic to other archaea [93, 94], for instance, of the Thermoproteota phylum [95], which is also present in the Laguna Lejía sediments.

The low relationship between the prokaryotic community structure and the geochemistry and mineralogy of sediment layers suggested that biological interactions (e.g*.*, symbiosis and competition) or abiotic variables not measured here (e.g*.*, moisture, temperature, redox potential, or physical structure of the sediment) could have played a decisive role on the microbial community structure of the Laguna Lejía terrace. Previous works on Atacama rocks showed that the physical structure better determined the microbial community composition and abundance than the chemistry of lithic substrates [83, 96]. There are, however, a few exceptions to the low relationship found here. Actinobacteriota, mainly represented in the Lejía terrace by the orders Gaiellales, Solirubrobacterales, and UBA5794, were positively correlated with the anorthoclase and anorthite feldspars (Table S5). To our knowledge, no previous studies have correlated Actinobacteriota with feldspars more than with other minerals. In fact, Gaiellales and Solirubrobacterales are widely distributed in extreme aquatic and terrestrial ecosystems with different substrates [97–99].

### The geochemistry of the *Laguna* Lejía terrace exerted selective pressure on microbial communities able to metabolize CO, S, N, As, and halogenated compounds

The prokaryotic community of the Lejía terrace is adapted to the particular geochemistry of the lake and shows a wide metabolic potential involved in S and N biogeochemical cycles. The high relative abundance of predicted enzymes for assimilatory and dissimilatory sulfate reduction, as well as sulfur and thiosulfate oxidation in all sediment layers (Fig. 4b) is consistent with the high SO_4_^2-^-S concentrations along the terrace profile (5–20 mg · g^-1^ dw). For instance, the detection of sulfate adenylyltransferases (e.g*.*, CysN) and sulfite reductases (e.g*.*, CysJ) (Table S6) suggested that microorganisms had the potential to transform sulfate to sulfide as a previous step for the synthesis of Cys amino acids (assimilatory) [100]. In addition, the sulfate adenylyltransferase (Sat), adenylyl-sulfate reductases (AprA and AprB), and sulfite reductases (DrsA and DsrB) suggested the potential of the prokaryotic community to first reduce sulfate to sulfite and then reduce sulfite to hydrogen sulfide (dissimilatory) [101]. In addition to sulfur reduction, the prokaryotic community also had six *sox* genes (*soxXYZABC*) that encode for a complete thiosulfate-oxidizing enzyme system [102]. This sulfur oxidation may be coupled, but not necessarily, to nitrate reduction in the Lejía prokaryotic community, based on the prediction of nitrate reductases (e.g*.*, NarG or NapA) [103]. In addition, the annotation of enzymes for nitrite reduction to ammonia (e.g*.*, NrfA), denitrification (e.g*.*, NirS, NirK, NorB, NosZ), and nitrification (AmoA, AmoB, AmoC, NxrA, and NxrB) [103, 104] suggested the potential of the prokaryotic community for a complete N cycle. This battery of nitrogen metabolisms possibly explains the bulk δ^15^N values from 1.6‰ to 6.1‰ in the sediments, as each nitrogen pathway is characterized by different levels of isotopic fractionations (*ɛ*), some higher (e.g., denitrification (*ɛ* = 28–33‰)) than others (e.g., ammonification (*ɛ* = 0–5‰)) [105].

The high relative abundance and wide distribution of predicted enzymes related to arsenic and halogenated compounds in the Lejía prokaryotic community (Fig. 4b) suggested that arsenic and halogenated compounds are important selective pressures for microorganisms. The annotation of enzymes involved in the arsenate (As(V)) reduction as a detoxification mechanism (e.g*.*, ArsA, ArsB, ArsC, ArsH, Acr3) in all sediment layers (Table S6) is consistent with the detection of As in the Lejía terrace profile (2–7 µg · g^-1^ dw). ArsC is involved in the reduction of arsenate to arsenite (As(III)), which is then pumped out of the cell with the efflux pumps ArsB and Acr3 [106, 107]. Also, the prediction of arsenite oxidases (AoxA and AoxB) suggested the potential of Lejía prokaryotes to oxidate arsenite to arsenate. The potential to transform arsenate or arsenite either as a detoxification mechanism or energy gain has also been reported in other Andean environments [70, 107, 108]. Unlike previous studies, the As cycle was surprisingly the most abundant metabolism in the Lejía prokaryotic community, reaching up to 27% of the total predicted proteins in sample D. In addition, the annotation of several haloalkane and haloacetate dehalogenases (DehH and DhaA) in the samples suggested the ability of the Lejía prokaryotic community to transform halogenated compounds [109].

The coexistence of aerobic and anaerobic carbon fixation pathways by the Lejía prokaryotic community indicated a variety of microconditions in the terrace and suggested autotrophic metabolism diversification to optimize available resources. The most abundant autotrophic metabolism was the photosynthetic assimilation of CO_2_ through the CBB cycle, based on the relative abundance of predicted Rubisco enzymes (RbcL and RbcS) (Table S6) [110]. Besides Cyanobacteria, other bacteria present in the Lejía sediments, such as α-, β-, and γ-Proteobacteria [110], may explain the dominance of the CBB cycle. The bulk carbon isotopic composition of the six Lejía samples (δ^13^C from −17‰ to −22‰), slightly more enriched in ^13^C than reported so far (δ^13^C from −27‰ to −29‰) [17], also supports the relevance of the CBB in the terrace. However, the slightly less negative values compared to the theoretical ones for the CBB cycle (δ^13^C from −20‰ to −38‰) [111, 112] suggested the contribution of other autotrophic pathways. For instance, these δ^13^C values could also imply a contribution from the reductive tricarboxylic acid (rTCA) cycle [113], but the absence of detection of any of the key genes associated with rTCA rules it out as a relevant CO_2_ fixation pathway in this environment. By contrast, the prediction of enzymes for the 3-HP/4-HB pathway (e.g*.*, 3-hydroxypropionyl-CoA dehydratase) in the lake terrace may explain the relatively ^13^C-enriched bulk δ^13^C, as the 3-HP/4-HB pathway produces characteristic bulk δ^13^C values from −0.2‰ to −4‰ [114]. In addition, the autotrophic community of the Laguna Lejía terrace was also involved in the WL pathway based on the detection of key enzymes, e.g*.*, anaerobic CO dehydrogenases [110].

Besides CO_2_ fixation, the high relative abundance of predicted CO dehydrogenases (CoxL, CoxM, and CoxS) in the Lejía prokaryotic community suggested aerobic CO oxidation as a determining trait for their survival in the organic carbon-depleted terrace. CO has been previously hypothesized to serve as a supplemental carbon and energy source in oligotrophic habitats [115]. The low TOC concentration in the Lejía terrace (0.1–1.2%) and the high relative abundance of predicted CO dehydrogenases form I (Figure S9) supported aerobic CO oxidation as a carbon and energy source in this organic carbon-depleted environment.

### Novelty of genomes from the *Laguna* Lejía terrace and expansion of metabolic capabilities to unexpected microbial taxa

The 591 MAGs reconstructed from the prokaryotic community of the Laguna Lejía terrace (Fig. 5) provided information on the metabolic potentials of yet uncultivated microorganisms from Andean ecosystems. The identification of bacterial MAGs not assigned to any known class, order, or family, as well as the large proportion of bacterial and archaeal MAGs unclassified to the genus (64% and 16%, respectively) or species (99.7% and 74%, respectively) level (Fig. 6), revealed the novelty of the recovered MAGs and evidenced the large amount of microbial dark matter in the Laguna Lejía ecosystem. For instance, the percentage of species novelty of the Lejía prokaryotic MAGs was 98.8% (considering bacteria and archaea together) and was higher than the 83% found in the Arctic ocean, calculated with the same methodology as here [52]. The absence of genomes in the GTDB similar to those recovered from the Lejía terrace showed up the need for microbial isolations from extreme environments and highlights the potential of omics techniques to gain insights into the functional diversity of uncultured microorganisms, expanding our view of the limits of life on Earth and providing biotechnological opportunities [1, 84].

The most frequent and widespread metabolic potentials among the Lejía MAGs were those related to the S and N cycles, aerobic CO oxidation, and transformation of As, Cu, and halogenated compounds. The presence of many of these metabolic capabilities among phylogenetically distant bacteria (Fig. 7) suggested gene acquisition by horizontal gene transfer. For instance, the ubiquitous presence of arsenic genes in the Lejía MAGs (i.e., all phyla except Cyanobacteria) and the reported location of *ars* operon in transposons [116] suggested horizontal gene transfer as a mechanism to facilitate bacterial adaptation to the particular Laguna Lejía chemical environment that requires in-depth study. Similarly, copper resistance proteins (Cop and Cus) may also be associated with mobile elements [117], and genes for haloalkane dehalogensases (DehH or DhaA) [109] and nitrate reductases (Nap and Nar) [118, 119] can be localized on plasmids and thus transfer laterally.

The broad redox capacity for N and S compounds as well as varied CO_2_ fixation pathways in phylogenetically distant bacteria and archaea from Laguna Lejía extends metabolic capabilities to previously unknown phyla. The prediction of potential nitrite oxidoreductases in phylogenetically distinct bacteria beyond Nitrospirota, Nitrospinota, Nitrosediminicolota, Chloroflexota, and Proteobacteria [120–122] may extend the list of nitrite oxidizers known so far. As the KEGG database does not differentiate between nitrite oxidoreductases and nitrate reductases (e.g*.*, K00370 and K00371), nitrite oxidation by these new phyla is to be considered with caution. Moreover, the prediction of ammonia monooxygenase (AmoC) in an unknown Actinobacteriota (class UBA4738, order AC-67) may expand the catalog of ammonia oxidizers, dominated in the Lejía samples by the archaeal family Nitrosopumilaceae (already described as ammonia oxidizers [123]) (Table S7). However, the sole presence of the *amoC* gene in the Actinobacteriota MAG does not ensure that ammonia oxidation occurs, as the rest of the *amo* gene cluster was missing (Figure S8).

While assimilatory sulfate reduction is frequent in all microorganisms, the dissimilatory pathway has only been described in Firmicutes, Proteobacteria (δ-Proteobacteria), Nitrospirae, and Euryarchaeota [124]. Therefore, the prediction of adenylylsulfate reductases in Bacteroidota and α- and γ-Proteobacteria in the Lejía MAGs may expand the list of dissimilatory sulfate reducers known so far. In addition, sulfur and thiosulfate oxidation was also widespread across MAGs, some belonging to already described phyla, such as Cyanobacteria or Proteobacteria (e.g*.*, Rhodospirillales or Pseudomonadales; Table S7) [125, 126], but also to potentially new taxa, such as Methylomirabilota (Rokubacteriales). The annotation of phosphoribulokinases (prk) in one Planctomycetota (Phycisphaerales) and Gemmatimonadota (order SG8-23), and a ribulose-bisphosphate carboxylase (Rubisco) in a Nanoarchaeota (Pacearchaeales), may also extend the list of microorganisms fixing CO_2_ through the CBB cycle known so far [127]. Alternatively, the prediction of these enzymes could be associated with a metabolism different from CBB, as occurs in the reductive hexulose-phosphase pathway mediated by Rubisco and prk in methanogenic archaea [128].

The expansion of genetic diversity and potential metabolisms involved in the biogeochemical cycles of carbon, nitrogen, and sulfur to unexpected microorganisms in the ancient sediments of the Laguna Lejía terrace contribute to our knowledge on the limits of life on Earth and on the habitability of similar present-day planetary settings. Our results demonstrate that bacterial and archaeal communities can thrive and adapt to changing environmental conditions over thousands—and perhaps millions—of years within a well-structured, slightly moist sediments. The microbial genetic and metabolic diversity in the terrace of the Laguna Lejía supports Martian craters that served as paleolake basins [129] as strategic landing sites for missions aimed at searching for signs of life, such the Mars 2020 mission, which landed in the Jezero crater delta to collect rock and regolith samples for possible return to Earth [130, 131].

## Conclusions

The microbial, geochemical and mineralogical analyses of the Laguna Lejía terrace (1 m section of ca. 10.3 and 11 kyr BP) showed:

i. A different structure of the prokaryotic community compared to that of the wet sediments reported in other studies, which suggests that lake desiccation provided a new ecological niche for microorganisms.
ii. A different prokaryotic community structure, geochemistry, and mineralogy between sediment layers, suggesting large-scale water level fluctuations in the Laguna Lejía during the 700 years that comprises the terrace section.
iii. Lipid biomarkers of aquatic/terrestrial plant remnants, as well as DNA from metabolically active microorganisms. The successful detection of biomarkers in the interior of the terrace supports Martian water basins (e.g*.*, craters) as strategic landing sites for seeking traces of life.
iv. A broad genetic and metabolic diversity of prokaryotic communities, capable of metabolizing CO, S, N, As, and halogenated compounds. This suggests that the particular geochemistry of the terrace (a saline, sulfur-rich, and organic carbon-depleted environment) exerted a selective pressure on the microbial communities.
v. A high number of novel bacterial and archaeal MAGs (e.g*.*, 98.8% belonged to currently undescribed species), as well as microbial taxa with hitherto undescribed metabolic capabilities. This represents a significant contribution to the expansion of microorganisms involved in the biogeochemical cycles of carbon, nitrogen, and sulfur.

## Supplementary Information

Supplementary Material 1.

## Data Availability

Raw DNA sequence reads and MAGs from the Laguna Lejía terrace were deposited at the NCBI Sequence Read Archive (SRA) and GenBank, and are available under the BioProject ID PRJNA1076829. Accessions for the raw DNA sequence reads are SAMN39953875-SAMN39953880, and for the genomes are SAMN39963127-SAMN39963352, and SAMN39963371-SAMN39963735. Additional information on radiocarbon data, lipid biomarker results, statistical analyses, prediction of metabolic potentials of the prokaryotic community and MAGs, and all raw data and intermediate files from the phylogenomics analysis can be found in the supplementary material.
