# Supplementary material for: Hyperexpansion of genetic diversity and metabolic capacity of extremophilic bacteria and archaea in ancient Andean lake sediments
Source: Microbiome. 2024 Sep 17;12:176. doi: 10.1186/s40168-024-01878-x (PMC11411797; doi:10.1186/s40168-024-01878-x)
Supplement: Supplementary file 2 — Supplementary Material 1. [file 40168_2024_1878_MOESM1_ESM.zip › Supplementary_Material_ESM.docx]

**Hyperexpansion of genetic diversity and metabolic capacity of extremophilic bacteria and archaea in ancient Andean lake sediments**

María Ángeles Lezcano^1,2*^, Till L.V. Bornemann^3,4^, Laura Sánchez-García^1^, Daniel Carrizo^1^, Panagiotis S. Adam^3,5^, Sarah P. Esser^3^, Nathalie A. Cabrol^6^, Alexander J. Probst^3,4^, Víctor Parro^1^

^1^ Centro de Astrobiología (CAB), CSIC-INTA, 28850 Torrejón de Ardoz, Madrid, Spain

^2^ IMDEA Water Institute, Avenida Punto Com 2, 28805 Alcalá de Henares, Madrid, Spain

^3^ Environmental Metagenomics, Research Center One Health Ruhr of the University Alliance Ruhr, Faculty of Chemistry, University of Duisburg-Essen, Essen, Germany

^4^ Centre of Water and Environmental Research (ZWU), University of Duisburg-Essen, Essen, Germany

^5^ Institute of General Microbiology, Kiel University, Kiel, Germany

^6^ SETI Institute, 339 Bernardo Avenue, Suite 200, Mountain View 94043, CA, USA

***Corresponding author:** María Ángeles Lezcano ([mangeles.lezcano@gmail.com](mailto:mangeles.lezcano@gmail.com), mangeles.lezcano@imdea.org)

**Table of contents**

**Text S1.** Gene arrangement and phylogenetic analyses of *amo* genes.

**Text S2.** Phylogenetic analysis of CO dehydrogenase forms I and II in the Lejía MAGs.

**Figure S1.** Conventional ^14^C dates for total organic fraction and reservoir-corrected age-depth relationship for samples A, C and F from the Laguna Lejía terrace.

**Figure S2.** Molecular distribution of lipids extracted in the non-polar fraction in the six sediment samples from the Laguna Lejía terrace.

**Figure S3.** Molecular distribution of the lipids extracted in the acidic fraction (alkanoic or fatty acids) in the six sediment samples from Laguna Lejía terrace.

**Figure S4.** Molecular distribution of the lipids extracted in the polar fraction in the six sediment samples from Laguna Lejía terrace.

**Figure S5.** Diversity (Shannon-Wiener index) and richness (number of different rpS3 genes) of Laguna Lejía sediment samples.

**Figure S6.** Principal Component Analysis (PCA) triplot of the prokaryotic community composition at the taxonomic phylum level of the Laguna Lejía sediments.

**Figure S7.** Principal coordinate analysis (PCoA) showing the dissimilarities of the Lejía sediment samples based on the Bray-Curtis index.

**Figure S8.** Gene arrangement showing the position and orientation of open reading frames up to 10 kbp upstream or downstream of *amo*ABC genes.

**Figure S9.** Maximum Likelihood phylogeny of the CoxL predicted in the bacterial MAGs from the Laguna Lejía terrace.

**Table S1.** Concentration of standards used in semiquantitative determination by ICP-MS.

**Table S2.** Relative abundance (coverage and % coverage) and number of rpS3 genes identified in the Laguna Lejía terrace at the kingdom, phylum and family level (**separated Excel file**).

**Table S3.** Key enzymes and encoding genes used for metabolic pathway annotations of assembled reads from the prokaryotic community and metagenome-assembled genomes (MAGs) from the Laguna Lejía terrace (**separated Excel file**).

**Table S4.** Pearson correlations between the abundance of phyla and soluble element concentrations in the sediments from the Laguna Lejía terrace.

**Table S5.** Pearson correlations between the abundance of phyla and minerals in the sediment samples from the Laguna Lejía terrace.

**Table S6.** Prediction of metabolic potencial of the prokaryotic community (metagenome assemblies) of the Laguna Lejía terrace based on the identification of key enzymes and encoding genes (**separated Excel file**).

**Table S7.** Map of predicted metabolic potential and estimated *in silico* replication rates of the 591 metagenome-assembled genomes (MAGs) reconstructed from the Laguna Lejía terrace (**separated Excel file**).

**File S1.** Phylogenetic tree of the *amo* genes of Archaea (**separated file**).

**File S2.** Phylogenetic tree of the *amo* genes of Bacteria (**separated file**).

**Text S1. Gene arrangement and phylogenetic analyses of *amo* genes.**

To verify that *amo* genes identified in the Lejía MAGs were not contamination from binning, synteny-based and phylogenetic analyses were performed to verify the origin of the *amo* genes. For gene arrangement analyses, genes starting or ending within 10 kbp on either side of an *amo*A, *amo*B or *amo*C gene identified on a scaffold were extracted. The position and orientation of *amo* genes and other open reading frames (ORF) were visualised and compared across genome bins using ggplot2 [1] and gggenes [2] (Figure S8). Gene arrangements were compared across closely related MAGs based on GTBD-tk taxonomy (Table S7). To verify the phylogenetic origin of *amo*ABC genes, two phylogenetic analyses were performed: for the bacterial MAG (*i.e.*, phylum Actinobacteriota, class UBA4738, genome ID Lejia_Bacteria_63_9) and for the archaeal MAGs. For the bacterial MAG, which only encoded for a single *amo*C gene on a 1,030 bp long scaffold, a single gene phylogeny was constructed (File S1). To this end, homolog sequences of *amo*C genes were identified in the UniRef100 database using the previously used *amo*C HMM model K10946 [3] with an e-value cutoff of 1e-5, pooled with bacterial and archaeal *amo*C genes identified in this study (Table S7), aligned with mafft-einsi [4], trimmed with BMGE [5] and the BLOSUM30 scoring matrix, and then phylogenetically evaluated with IQ-TREE2 [6] and the “"-m MFP -mset JTT,WAG,LG -mfreq FU,F,FO -bb 1000 -alrt 1000” options. For the archaeal MAGs, which all contained at least two of the three *amo* genes (*i.e.*, *amo*A, *amo*B and/or *amo*C), a concatenated gene phylogeny was performed (File S2). The WhereDoGGo pipeline v20240621 (https://github.com/MEDEAlab/WhereDoGGo) was used, using the *amo*ABC HMM models [3] with a score cutoff of 40 (corresponding to an approximate e-value cutoff of 1e-5) with the default parameters and the IQ-TREE command described above. The reference genomes used in WhereDoGGo were those classified as p__Thermoproteota in GTDB r220 [7], as all archaeal genomes in this study belong to this phylum. GTDB-tk classifications of Lejía MAGs were compared to reference taxonomy classifications in the phylogenetic trees to check for possible contamination.

**Text S2. Phylogenetic analysis of CO dehydrogenase forms I and II in the Lejía MAGs.**

To classify aerobic CO dehydrogenase (CODH) homologs in the Lejía MAGs we extracted sequences annotated as the large (CoxL; K03520), medium (CoxM; K03519), and small subunits (CoxS; K03518), and we then focused on CoxL. The coxL sequences of Lejía genomes were spiked i) with sequences in the Swiss-Prot database (except those from *Escherichia coli*) containing a cross-reference to Pfam entry PF01315 (*i.e.*, Aldehyde oxidase and xanthine dehydrogenase, a/b hammerhead domain), and ii) sequences corresponding to CODH form I and II (or the most similar hits in Uniprot) from *Roseibium aggregatum* (*Stappia aggregata*) and *Ruegeria pomeroyi* (*Silicibacter pomeroyi*) from King (2003) [8]. We aligned sequences with MAFFT E-INS-i 7.526 [4], trimmed with BMGE version 1.12 [5], and constructed a maximum-likelihood phylogeny with IQ-TREE 2.3.4 [6] with the model automatically selected by Modelfinder [9] out of the WAG, JTT, and LG replacement matrices (-m MFP -mset WAG,JTT,LG). The branch supports were converted to 1/0 (strongly/weakly supported, where strongly is UFBOOT>=95 and aLRT SH-like >=80) with the fixdualsupports.py script included in WhereDoGGo pipeline v20240621 (https://github.com/MEDEAlab/WhereDoGGo). We visualized the tree with iTOL version 6.9.1 [10]. A sequence for the conserved motif of putative CODH form II was built by extracting the sequences corresponding to said branches, realigning as above, trimming the alignment on both terminals and using WebLogo 3 [11]. All raw data are included as supplementary material.


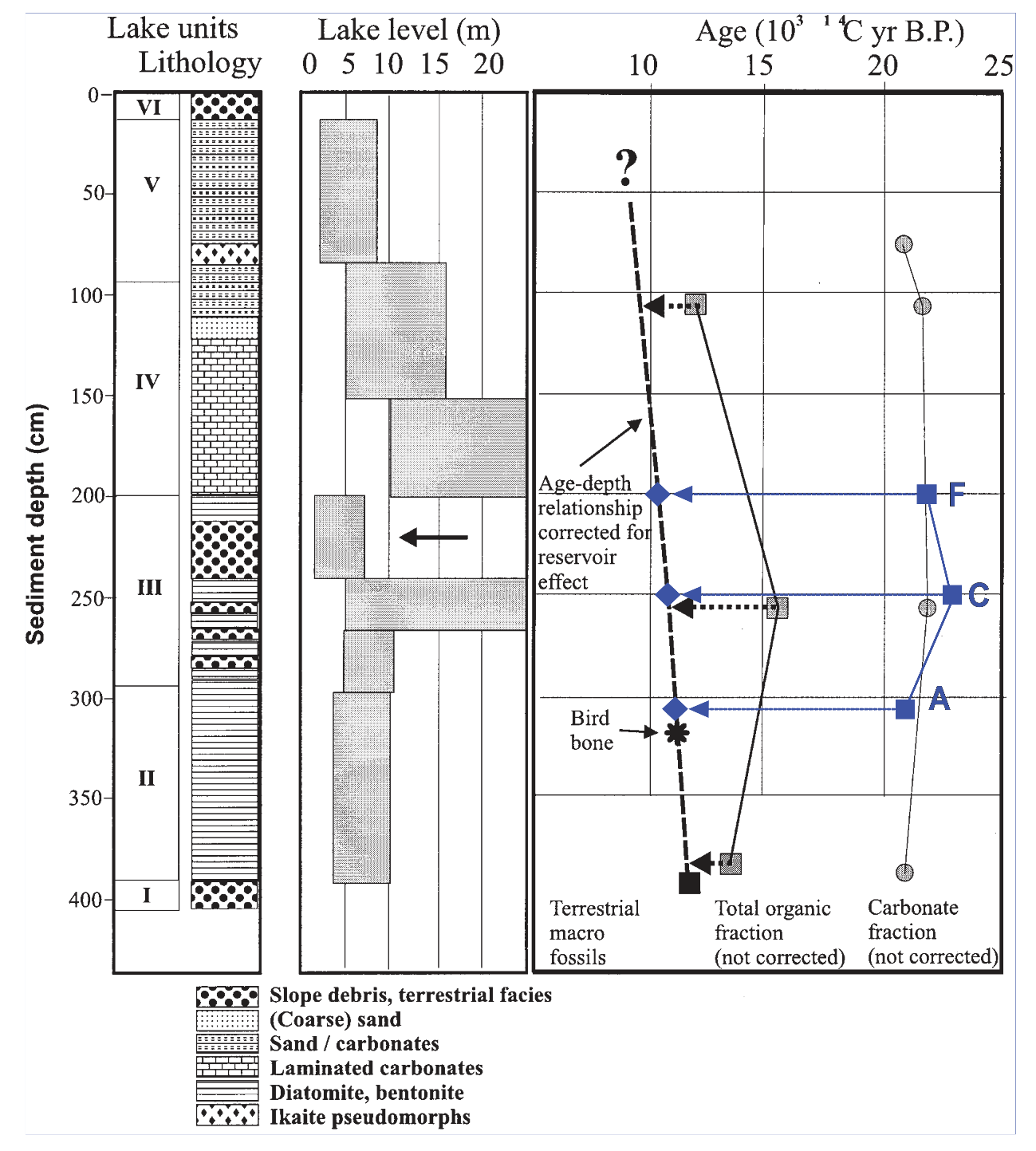


**Figure S1.** Conventional ^14^C dates for total organic fraction (blue squares) and reservoir-corrected age-depth relationship (blue diamonds on the dashed black line) for samples A, C and F from the Laguna Lejía terrace based on data calculations from Geyh et al. (1999) (dark/grey circles and squares). Reservoir-corrected age of sample A is ~11,000 yr BP, that of sample C is ~10,650 yr BP, and that of sample F is ~10,300 yr BP. The figure used as the basis for our calculations is extracted from Geyh et al. (1999) (Cambridge University Press, reproduced with permission).

**Figure S2**. Molecular distribution of lipids extracted in the non-polar fraction in the six sediment samples from Laguna Lejía terrace: linear and saturated alkanes (*n*-alkanes or *n*-C_n_), alkanes with one double bond (alkenes or C_n:1_), and isoprenoids (squalene and phytane). The dashed lines separate the distinct groups of lipid families.

**Figure S3**. Molecular distribution of the lipids extracted in the acidic fraction (alkanoic or fatty acids) in the six sediment samples from Laguna Lejía terrace: straight chain fatty acids (*n*-fatty acids), fatty acids with a methyl group in the N-1 (*iso* or *-i*) or N-2 (*anteiso* or *a-*) carbon, other monomethylated fatty acids (e.g., 10Me-C_16:0_), tetramethylated (i.e., 3,7,11,15Me-C_16:0_) fatty acids, monounsaturated fatty acids (e.g., C_16:1_), cyclopropyl fatty acids (i.e., Cy-C_19:0_), and hopanoic acid (i.e., 17β(H),21β(H)-bishomohopanoic acid or ββ-C_32_-hopanoic acid). The dashed lines separate the distinct groups of lipid families.

**Figure S4.** Molecular distribution of the lipids extracted in the polar fraction in the six sediment samples from Laguna Lejía terrace: *n*-alkanols, alkanodiols, phytol, and sterols. The dashed lines separate the distinct groups of lipid families.

**Figure S5.** Diversity (Shannon-Wiener index) and richness (number of different rpS3 genes) of Laguna Lejía sediment samples.

**Figure S6.** Principal Component Analysis (PCA) triplot of the prokaryotic community composition at the taxonomic phylum level (black triangles) of the Laguna Lejía sediments (blue dots). Soluble elements (red vectors) and minerals (red vectors) with high positive (r>0.8) or negative (r<-0.8) correlation with phyla (Table S4 and S5) were *post hoc* included in the PCA as supplementary variables to aid interpretation. Labels from prokaryotes are: Acido, Acidobacteriota; Actino, Actinobacteriota; Arma, Armatimonadota; Bacte, Bacteroidota; Bdello, Bdellovibrionota; Calda, Caldatribacteriota; Chlorofl, Chloroflexota; Cyano, Cyanobacteria; Deino, Deinococcota; Depen, Dependentiae; Desul, Desulfobacterota; Eisen, Eisenbacteria; Firmi, Firmicutes; Gem, Gemmatimonadota; Hydro, Hydrogenedentota; Methy, Methylomirabilota; Myxo, Myxococcota, Nanoar, Nanoarchaeota; Nitros, Nitrospirota; Pates, Patescibacteria; Planc, Planctomycetota; Proteo, Proteobacteria; Spiro, Spirochaetota; Thermo, Thermoproteota; Verru, Verrucomicrobiota; Zixi, Zixibacteria.

**Figure S7.** Principal coordinate analysis (PCoA) showing the dissimilarities of the Lejía sediment samples (black dots) based on the Bray-Curtis index calculated from the relative abundances of the rpS3 genes.

**Figure S8**. Gene arrangement showing the position and orientation of open reading frames up to 10 kbp upstream or downstream of *amo*ABC genes in each scaffold. The *amo*A, *amo*B and *amo*C genes are shown in green, yellow and purple, respectively, while other non-*amo* genes are grouped as ‘Other’ in grey. To facilitate a common x-axis, the start of each synteny block was set to 0. The y-axis shows the genome ID and the lowest taxonomy level (order, family, genus or species) of each MAG according to GTDB-tk [13] (Table S7).

**Figure S9.** Maximum Likelihood phylogeny (model selected in IQ-TREE by Modelfinder) of the predicted CoxL in the bacterial MAGs from the Laguna Lejía terrace, CoxL sequences with form I and II present in the Swiss-Prot database, and sequences containing a cross-reference to Pfam PF01315 entry (*i.e.*, Aldehyde oxidase and xanthine dehydrogenase, a/b hammerhead domain). All clades have been collapsed to simplify the tree except for the sequences that comprises the CODH form I cluster. The name of the sequences refers to the name of the scaffold in which the CoxL protein has been predicted, followed by a hyphen and the Genome ID of each MAG according to GTDB-tk (Table S7).

| Element | External Standard (ppb) | QC.Std (ppb) |
| --- | --- | --- |
| Ag | 50 | 100 |
| Al | 49 | 98 |
| As | 490 | 980 |
| B | 490 | 980 |
| Ba | 48.5 | 97 |
| Be | 510 | 1020 |
| Bi | 49 | 98 |
| Ca | 4945 | 9890 |
| Cd | 49.5 | 99 |
| Co | 49.5 | 99 |
| Cr | 49.5 | 99 |
| Cu | 49.5 | 99 |
| Fe | 500 | 1000 |
| Ga | 49.5 | 99 |
| K | 49 | 98 |
| Li | 49.5 | 99 |
| Mg | 49 | 98 |
| Mn | 49.5 | 99 |
| Mo | 49.5 | 99 |
| Na | 48.5 | 97 |
| Ni | 49.5 | 99 |
| Pb | 49 | 98 |
| Rb | 49.5 | 99 |
| Se | 505 | 1010 |
| Sr | 500 | 1000 |
| Te | 49.5 | 99 |
| Tl | 49.5 | 99 |
| U | 49 | 98 |
| V | 49 | 98 |
| Zn | 495 | 990 |
| Ce | 50 | 100 |
| Dy | 50 | 100 |
| Er | 50 | 100 |
| Eu | 50 | 100 |
| Gd | 50 | 100 |
| Ho | 50 | 100 |
| La | 50 | 100 |
| Lu | 50 | 100 |
| Nd | 50 | 100 |
| Pr | 50 | 100 |
| Sm | 50 | 100 |
| Sc | 50 | 100 |
| Tb | 50 | 100 |
| Th | 50 | 100 |
| Tm | 50 | 100 |
| Y | 50 | 100 |
| Yb | 50 | 100 |

**Table S1.** Concentration of standards used in semiquantitative determination by ICP-MS.

**Table S4.** Pearson correlations between the abundance of phyla (calculated as the sum of rpS3 coverage) and soluble element concentrations in the sediments from the Laguna Lejía terrace. Red numbers indicate statistically significant positive correlations (r>0.8, *p*-value<0.05), and blue numbers indicate statistically significant negative correlations (r<-0.8, *p*-value<0.05).

| Phyla | Cl | Na | Mg | K | Ca | SO_4_^3-^-S | B | F | Li | NO_3_^-^-N | Br | Ar | V | Rb | Sr | Mo | Bi |
| --- | --- | --- | --- | --- | --- | --- | --- | --- | --- | --- | --- | --- | --- | --- | --- | --- | --- |
| Acidobacteriota | -0.30 | -0.39 | -0.55 | 0.00 | 0.00 | -0.75 | -0.36 | -0.28 | -0.34 | -0.08 | -0.07 | 0.39 | 0.13 | -0.12 | 0.07 | -0.53 | -0.19 |
| Actinobacteriota | -0.67 | -0.69 | -0.65 | -0.28 | 0.10 | **-0.85** | -0.62 | -0.69 | -0.62 | -0.61 | -0.40 | -0.22 | 0.04 | -0.53 | -0.07 | -0.68 | -0.24 |
| Armatimonadota | -0.45 | -0.36 | -0.25 | -0.28 | -0.37 | -0.63 | -0.10 | -0.32 | -0.36 | -0.57 | -0.52 | 0.09 | 0.30 | -0.52 | -0.44 | -0.27 | 0.29 |
| Bacteroidota | -0.05 | -0.22 | -0.31 | -0.18 | 0.35 | -0.01 | -0.46 | -0.01 | -0.23 | 0.14 | 0.17 | -0.35 | -0.53 | 0.07 | 0.26 | -0.27 | -0.70 |
| Bdellovibrionota | -0.31 | -0.46 | -0.61 | 0.22 | **0.98** | -0.24 | -0.78 | -0.68 | -0.30 | 0.03 | 0.30 | -0.47 | -0.39 | 0.12 | **0.81** | -0.64 | -0.70 |
| Caldatribacteriota | -0.45 | -0.36 | -0.25 | -0.28 | -0.37 | -0.63 | -0.10 | -0.32 | -0.36 | -0.57 | -0.52 | 0.09 | 0.30 | -0.52 | -0.44 | -0.27 | 0.29 |
| Chloroflexota | -0.70 | -0.81 | **-0.94** | -0.37 | 0.38 | **-0.89** | -0.75 | -0.51 | -0.77 | -0.33 | -0.34 | -0.09 | -0.58 | -0.43 | 0.13 | **-0.91** | -0.22 |
| CSP1-3 | -0.28 | -0.16 | 0.07 | -0.24 | 0.30 | 0.26 | -0.12 | -0.33 | -0.15 | -0.44 | -0.29 | -0.78 | -0.33 | -0.33 | -0.04 | 0.01 | 0.18 |
| Cyanobacteria | -0.18 | -0.20 | -0.19 | 0.26 | **0.85** | 0.16 | -0.44 | -0.61 | -0.05 | -0.10 | 0.22 | -0.65 | -0.20 | 0.09 | 0.64 | -0.26 | -0.34 |
| Deinococcota | 0.30 | 0.22 | 0.22 | -0.19 | -0.37 | 0.22 | 0.11 | 0.46 | 0.11 | 0.20 | 0.12 | 0.03 | -0.01 | 0.13 | -0.23 | 0.27 | -0.40 |
| Dependentiae | 0.32 | 0.36 | 0.30 | -0.04 | -0.34 | 0.31 | 0.57 | 0.68 | 0.24 | 0.39 | -0.02 | 0.51 | -0.24 | 0.16 | -0.25 | 0.35 | 0.65 |
| Desulfobacterota | 0.12 | 0.21 | 0.31 | -0.31 | -0.22 | 0.44 | 0.40 | 0.48 | 0.07 | 0.05 | -0.24 | -0.09 | -0.49 | -0.11 | -0.35 | 0.34 | 0.58 |
| Eisenbacteria | 0.28 | 0.08 | -0.10 | 0.29 | 0.25 | 0.05 | -0.28 | 0.03 | 0.14 | 0.42 | 0.56 | 0.05 | 0.11 | 0.45 | 0.46 | -0.07 | **-0.89** |
| Firmicutes | -0.38 | -0.33 | -0.23 | -0.36 | -0.48 | -0.61 | -0.12 | -0.18 | -0.36 | -0.50 | -0.47 | 0.10 | 0.27 | -0.49 | -0.51 | -0.23 | 0.10 |
| Gemmatimonadota | -0.40 | -0.37 | -0.31 | -0.32 | -0.41 | -0.68 | -0.17 | -0.22 | -0.39 | -0.46 | -0.44 | 0.17 | 0.25 | -0.46 | -0.43 | -0.31 | 0.08 |
| Hydrogenedentota | -0.45 | -0.36 | -0.25 | -0.28 | -0.37 | -0.63 | -0.10 | -0.32 | -0.36 | -0.57 | -0.52 | 0.09 | 0.30 | -0.52 | -0.44 | -0.27 | 0.29 |
| Methylomirabilota | -0.52 | -0.43 | -0.31 | -0.32 | -0.31 | -0.67 | -0.18 | -0.40 | -0.43 | -0.63 | -0.55 | 0.01 | 0.25 | -0.57 | -0.42 | -0.33 | 0.25 |
| Myxococcota | -0.45 | -0.35 | -0.26 | -0.34 | -0.44 | -0.66 | -0.04 | -0.21 | -0.38 | -0.53 | -0.56 | 0.20 | 0.22 | -0.54 | -0.51 | -0.27 | 0.41 |
| Nanoarchaeota | 0.16 | 0.18 | 0.08 | -0.15 | -0.38 | 0.04 | 0.42 | 0.60 | 0.05 | 0.30 | -0.14 | 0.58 | -0.30 | 0.03 | -0.31 | 0.15 | 0.62 |
| Nitrospirota | 0.76 | 0.81 | **0.82** | 0.12 | -0.76 | 0.69 | **0.90** | **0.98** | 0.66 | 0.53 | 0.22 | 0.59 | 0.32 | 0.40 | -0.43 | **0.87** | 0.40 |
| Patescibacteria | -0.41 | -0.42 | -0.44 | -0.51 | -0.47 | -0.74 | -0.16 | 0.03 | -0.51 | -0.33 | -0.51 | 0.31 | -0.14 | -0.48 | -0.52 | -0.39 | 0.17 |
| Planctomycetota | -0.62 | -0.62 | -0.45 | -0.66 | 0.34 | -0.21 | -0.59 | -0.43 | -0.65 | -0.61 | -0.51 | **-0.91** | -0.78 | -0.60 | -0.13 | -0.46 | -0.20 |
| Proteobacteria | 0.17 | 0.23 | 0.23 | -0.33 | -0.75 | 0.01 | 0.57 | 0.73 | 0.05 | 0.14 | -0.31 | 0.62 | -0.13 | -0.12 | -0.65 | 0.30 | 0.70 |
| Spirochaetota | 0.10 | 0.02 | 0.07 | -0.31 | -0.21 | 0.12 | -0.10 | 0.23 | -0.07 | 0.00 | 0.00 | -0.25 | -0.15 | -0.04 | -0.21 | 0.11 | -0.49 |
| Thermoproteota | -0.11 | -0.28 | -0.53 | 0.47 | **0.91** | -0.23 | -0.61 | -0.54 | -0.10 | 0.29 | 0.50 | -0.08 | -0.16 | 0.35 | **0.91** | -0.55 | -0.64 |
| Verrucomicrobiota | 0.23 | 0.05 | -0.20 | 0.59 | **0.83** | 0.19 | -0.36 | -0.22 | 0.20 | 0.56 | 0.72 | -0.05 | -0.12 | 0.61 | **0.93** | -0.21 | -0.69 |
| Zixibacteria | -0.27 | -0.37 | -0.55 | -0.04 | 0.57 | -0.26 | -0.40 | -0.19 | -0.33 | 0.13 | 0.02 | -0.03 | -0.68 | 0.00 | 0.40 | -0.52 | -0.05 |

**Table S5.** Pearson correlations between the abundance of phyla (calculated as the sum of rpS3 coverage) and minerals in the sediment samples from the Laguna Lejía terrace. Red numbers indicate statistically significant positive correlations (r>0.8, p-value<0.05), and blue cells indicate statistically significant negative correlations (r<-0.8, *p*-value<0.05).

| Phyla | Magnesium Calcite | Albite | Andesine | Anorthite | Anorthoclase | Muscovite | Gypsum | Halite | Quartz |
| --- | --- | --- | --- | --- | --- | --- | --- | --- | --- |
| Acidobacteriota | -0.11 | 0.07 | -0.70 | **0.84** | 0.66 | 0.02 | -0.54 | -0.06 | 0.03 |
| Actinobacteriota | -0.42 | -0.42 | -0.18 | **0.89** | **0.82** | -0.40 | -0.10 | -0.55 | 0.14 |
| Armatimonadota | -0.46 | -0.27 | -0.27 | **0.87** | **1.00** | -0.23 | -0.28 | -0.37 | -0.43 |
| Bacteroidota | 0.29 | 0.22 | 0.08 | -0.49 | -0.61 | 0.01 | 0.22 | 0.07 | **0.83** |
| Bdellovibrionota | -0.04 | -0.30 | 0.30 | 0.00 | -0.40 | -0.17 | 0.42 | -0.34 | 0.77 |
| Caldatribacteriota | -0.46 | -0.27 | -0.27 | **0.87** | **1.00** | -0.23 | -0.28 | -0.37 | -0.43 |
| Chloroflexota | -0.50 | 0.26 | -0.17 | 0.45 | 0.26 | 0.34 | 0.13 | -0.49 | 0.26 |
| CSP1-3 | -0.41 | -0.40 | **0.98** | -0.44 | -0.30 | -0.18 | **0.87** | -0.55 | 0.00 |
| Cyanobacteria | -0.12 | -0.58 | 0.73 | -0.20 | -0.43 | -0.35 | 0.67 | -0.42 | 0.44 |
| Deinococcota | 0.53 | 0.25 | -0.28 | -0.37 | -0.25 | -0.13 | -0.29 | 0.45 | 0.41 |
| Dependentiae | -0.03 | **0.82** | -0.11 | -0.50 | -0.36 | **0.90** | -0.02 | 0.35 | -0.56 |
| Desulfobacterota | -0.20 | 0.54 | 0.49 | **-0.81** | -0.55 | 0.65 | 0.51 | 0.02 | -0.37 |
| Eisenbacteria | 0.69 | -0.05 | -0.42 | -0.06 | -0.31 | -0.36 | -0.39 | 0.43 | **0.82** |
| Firmicutes | -0.27 | -0.17 | -0.41 | 0.77 | **0.92** | -0.28 | -0.40 | -0.22 | -0.24 |
| Gemmatimonadota | -0.29 | -0.13 | -0.47 | **0.83** | **0.93** | -0.21 | -0.43 | -0.22 | -0.23 |
| Hydrogenedentota | -0.46 | -0.27 | -0.27 | **0.87** | **1.00** | -0.23 | -0.28 | -0.37 | -0.43 |
| Methylomirabilota | -0.50 | -0.32 | -0.21 | **0.88** | **1.00** | -0.27 | -0.21 | -0.45 | -0.38 |
| Myxococcota | -0.50 | -0.08 | -0.33 | **0.83** | **0.98** | -0.03 | -0.30 | -0.34 | -0.53 |
| Nanoarchaeota | -0.14 | **0.91** | -0.28 | -0.29 | -0.17 | **0.97** | -0.12 | 0.27 | -0.55 |
| Nitrospirota | 0.53 | 0.53 | -0.35 | -0.52 | -0.27 | 0.32 | -0.46 | 0.78 | -0.43 |
| Patescibacteria | -0.33 | 0.42 | -0.57 | 0.55 | 0.68 | 0.31 | -0.38 | -0.13 | -0.23 |
| Planctomycetota | -0.49 | -0.11 | 0.79 | -0.40 | -0.28 | -0.04 | **0.88** | -0.67 | 0.43 |
| Proteobacteria | -0.11 | **0.87** | -0.40 | -0.18 | 0.10 | **0.81** | -0.30 | 0.32 | -0.68 |
| Spirochaetota | 0.38 | 0.09 | -0.05 | -0.37 | -0.25 | -0.25 | -0.05 | 0.21 | 0.54 |
| Thermoproteota | 0.12 | -0.18 | -0.04 | 0.17 | -0.31 | -0.07 | 0.08 | -0.10 | 0.63 |
| Verrucomicrobiota | 0.44 | -0.08 | 0.01 | -0.24 | -0.69 | -0.06 | 0.08 | 0.20 | 0.72 |
| Zixibacteria | -0.29 | 0.46 | 0.14 | -0.21 | -0.44 | 0.66 | 0.39 | -0.21 | 0.19 |

**References**

1. Wickham H. ggplot2: Elegant Graphics for Data Analysis. New York: Springer-Verlag; 2016.

2. Wilkins D. _gggenes: Draw Gene Arrow Maps in ’ggplot2’_. R package version 0.4.1. 2020. https://cran.r-project.org/package=gggenes

3. Aramaki T, Blanc-Mathieu R, Endo H, Ohkubo K, Kanehisa M, Goto S, et al. KofamKOALA: KEGG Ortholog assignment based on profile HMM and adaptive score threshold. Valencia A, editor. Bioinformatics. 2020;36:2251–2.

4. Katoh K, Standley DM. MAFFT Multiple Sequence Alignment Software Version 7: Improvements in Performance and Usability. Mol Biol Evol. 2013;30:772–80.

5. Criscuolo A, Gribaldo S. BMGE (Block Mapping and Gathering with Entropy): a new software for selection of phylogenetic informative regions from multiple sequence alignments. BMC Evol Biol. 2010;10:210.

6. Minh BQ, Schmidt HA, Chernomor O, Schrempf D, Woodhams MD, von Haeseler A, et al. IQ-TREE 2: New Models and Efficient Methods for Phylogenetic Inference in the Genomic Era. Teeling E, editor. Mol Biol Evol. 2020;37:1530–4.

7. Parks DH, Chuvochina M, Waite DW, Rinke C, Skarshewski A, Chaumeil P-A, et al. A standardized bacterial taxonomy based on genome phylogeny substantially revises the tree of life. Nat Biotechnol. 2018;36:996–1004.

8. King GM. Molecular and Culture-Based Analyses of Aerobic Carbon Monoxide Oxidizer Diversity. Appl Environ Microbiol. 2003;69:7257–65.

9. Kalyaanamoorthy S, Minh BQ, Wong TKF, von Haeseler A, Jermiin LS. ModelFinder: fast model selection for accurate phylogenetic estimates. Nat Methods. 2017;14:587–9.

10. Letunic I, Bork P. Interactive Tree of Life (iTOL) v6: recent updates to the phylogenetic tree display and annotation tool. Nucleic Acids Res. 2024;52:W78–82.

11. Crooks GE, Hon G, Chandonia J-M, Brenner SE. WebLogo: A Sequence Logo Generator. Genome Res. 2004;14:1188–90.

12. Geyh MA, Grosjean M, Núñez L, Schotterer U. Radiocarbon Reservoir Effect and the Timing of the Late-Glacial/Early Holocene Humid Phase in the Atacama Desert (Northern Chile). Quat Res. 1999;52:143–53.

13. Chaumeil P-A, Mussig AJ, Hugenholtz P, Parks DH. GTDB-Tk: a toolkit to classify genomes with the Genome Taxonomy Database. Hancock J, editor. Bioinformatics. 2019;36:1925–7.
